# The BHARAT study: a multi-modal, multi-omics investigation of aging signatures in the Indian population

**DOI:** 10.18632/aging.206373

**Published:** 2026-04-24

**Authors:** Suramya Asthana, Amruth Deepak Bhat, Gayathri Mahadevan, Lakshmi Kothegala, Swati Negi, Seema Yadav, Anupama Sudhakaran, Annwesha Roy, Meet Makwana, Rahul Patel, Harshavardhan Rao B, Caroline Elizabeth George, Srimonta Gayen, Shantanu Shukla, Siddharth Jhunjhunwala, Prosenjit Sen, Prabhdeep Kaur, Ramray Bhat, Deepak Kumar Saini, on behalf of the Longevity India Team, Collaborators

**Affiliations:** 1 Longevity India, Division of Interdisciplinary Sciences, Indian Institute of Science, Bengaluru 560012, Karnataka, India; 2 Department of Developmental Biology and Genetics, Division of Biological Sciences, Indian Institute of Science, Bengaluru 560012, Karnataka, India; 3 Department of Bioengineering, Division of Interdisciplinary Sciences, Indian Institute of Science, Bengaluru 560012, Karnataka, India; 4 Department of Gastroenterology, MS Ramaiah Medical College, RUAS, Bengaluru 560054, Karnataka, India; 5 Community Health, Palliative Care and Research Division, Bangalore Baptist Hospital, Bengaluru 560032, Karnataka, India; 6 Centre for Nano Science and Engineering, Division of Interdisciplinary Sciences, Indian Institute of Science, Bengaluru 560012, Karnataka, India; 7 Isaac Centre for Public Health, Tata IISc Medical School, Indian Institute of Science, Bengaluru 560012, Karnataka, India

**Keywords:** aging, protocol, multi-omics, population study, biomarker discovery

## Abstract

India is undergoing a rapid demographic transition, with its elderly population projected to exceed 347 million by 2050. Although aging is the primary risk factor for multiple chronic diseases, most biological age (BA) models have been developed for Western populations, with limited applicability to Indian demographics. The BHARAT study (Biomarkers of Healthy Aging, Resilience, Adversity, and Transitions) aims to develop and validate composite signatures of aging in the Indian population by integrating multi-omics, biochemical, clinical, and lifestyle data. The BHARAT study is a multi-center, cross-sectional observational study designed using a hub-and-spoke model, with the Indian Institute of Science (IISc) serving as the central hub for omics analyses, biobanking, and data integration. Participants are stratified into five age groups (18–29, 30–44, 45–59, 60–74, ≥75 years) with balanced rural–urban and gender representation. The study primarily includes healthy participants, excluding those with chronic diseases that are not resolved by medication. Data collection encompasses comprehensive clinical and cognitive assessments, lifestyle and quality-of-life questionnaires, and biological sampling (including blood, urine, stool, cheek swabs, and hair). Multi-omics profiling spans epigenomics, proteomics, metabolomics, lipidomics, metagenomics, and immune phenotyping, integrating untargeted discovery-based Liquid Chromatography-Tandem Mass Spectrometry (LC-MS/MS) with targeted assays under harmonized protocols and quality-controlled biobanking standards. As the first large-scale, discovery-driven aging cohort in India, BHARAT will generate population-specific reference datasets, (re)train and calibrate biological clocks, develop a data-driven framework for organ-specific clocks, and identify biomarkers of physiological resilience and decline. Given that presently this study is cross-sectional in design, it will help establish a scalable framework for subsequent longitudinal and translational research to develop context-specific diagnostics, predictive models, and therapeutic targets for healthy aging in India.

## INTRODUCTION

The global demographic transition toward aging populations poses a significant challenge for healthcare systems and societies [1, 2]. While life expectancy has steadily increased over the past century, the extension of the healthspan, the period of life free from chronic disease and functional decline, has remained unchanged. This widening gap between healthspan and lifespan contributes to morbidity, increased healthcare expenditure, and a reduced quality of life among older adults [3–6].

A key factor in this disparity is the difference between chronological age, a measure of elapsed lifetime, and biological age, which reflects the cumulative physiological experience of genetic, molecular, and environmental factors [7, 8]. Unlike chronological age, biological age (BA) can serve as a dynamic indicator of functional capacity, disease risk, and resilience reserve, which are much better predictors for all-cause mortality in the elderly [9–13]. However, to date, most aging studies have focused on single-omics datasets or narrowly targeted biomarker panels [14–27]. While these approaches have led to the identification of candidate biomarkers of aging, their correlations remain weak, and no standardized or universally validated biomarkers of aging currently exist - a persistent challenge in the field [28–30]. This limitation is partly due to the compartmentalized nature of earlier technologies, which were unable to comprehensively capture the multidimensional biological processes underlying aging. Advances in the quantification of BA, have opened new avenues for risk stratification and personalized interventions [31–34].

Recent technological advances have enabled the generation of high-quality, large-scale multi-omics datasets that encompass profiles of various biological samples across multiple domains, including genomics, transcriptomics, proteomics, metabolomics, lipidomics, and metagenomics [35–39]. This marks a critical inflection point in aging research, and for the first time, we possess the capability to interrogate aging as a systems-level process across multiple molecular and physiological levels in population-scale studies. Furthermore, the advent of artificial intelligence (AI) and large language model (LLM)-driven analytics provides unprecedented computational power to mine, interpret, and integrate these complex datasets [38, 40–44]. These tools enable the development of untargeted, data-driven pipelines for biomarker discovery, multidimensional modelling, and prediction of biological resilience in large cohorts. Cohort studies worldwide have begun employing these integrative multi-omics and AI-driven approaches to unravel the complexity of aging [45–48]. However, most existing datasets that use these analytical frameworks are predominantly derived from Western populations, leaving Asia, and particularly Indian populations, significantly underrepresented in global aging research [49, 50].

Interestingly, India presents a distinctive scientific opportunity in this regard, with an elderly population projected to reach 347 million by 2050, the country faces an urgent need to identify context-specific biomarkers of aging [51]. However, the country currently lacks comprehensive, high-quality reference datasets for aging and healthspan assessment. Clinical benchmarks and diagnostic cut-offs continue to rely heavily on data derived from Western cohorts, which may not accurately capture the physiological and molecular baselines of Indian populations [52, 53]. Moreover, the unique features of aging that may emerge from India’s extraordinary diversity in genetic ancestry, diet, lifestyle, environmental exposures, and rural-urban heterogeneity remain entirely unexplored [54–58]. These gaps highlight the critical need for a large-scale, integrative study to define India-specific signatures of biological aging and resilience, thereby enabling more precise, equitable, and population-relevant approaches to healthy aging.

To address these gaps, the Longevity India initiative was established to integrate cross-disciplinary expertise in aging biology, clinical research, and technology development [59]. Within this framework, the BHARAT Study (Biomarkers of Healthy Aging, Resilience, Adversity, and Transitions) has been designed as a large-scale, multi-modal, multi-parametric, and multi-omics cohort investigation. The study brings together researchers, clinicians, industry partners, and communities to generate high-quality data and address key questions related to healthy aging. By capturing the breadth of India’s demographic and environmental diversity, the BHARAT study aims to define biological age, resilience, and context-specific biomarkers that can inform predictive models and translational strategies for aging research.

## Objectives

### Primary objective

- Develop and validate composite signatures of biological aging using cellular, molecular, clinical, and lifestyle metrics.

### Secondary objectives

- Identify resilience- and frailty-associated biomarkers.
- Establish a validated frailty index tailored for Indian cohorts.
- Build AI/ML-based predictive models for BA; disease risk and all-cause mortality.
- Establish a scalable Indian biobank for aging research.
- Establish baseline values for various biological features for India

### Study team and collaborative framework

The BHARAT study is conducted under the framework of the Longevity India initiative, which comprises a multi-institutional, cross-disciplinary network of investigators from biological sciences, clinical medicine, engineering, and computational sciences.

The BHARAT Study is designed as a cross-sectional cohort study conceptualized between 2023-24, established partners, obtained ethical approvals in 2024 and initiated population sampling in 2025. The study employs a hub-and-spoke model in which IISc serves as the central hub while multiple clinical partners act as regional nodes for participant recruitment and primary data collection (Figure 1).

**Figure 1:**
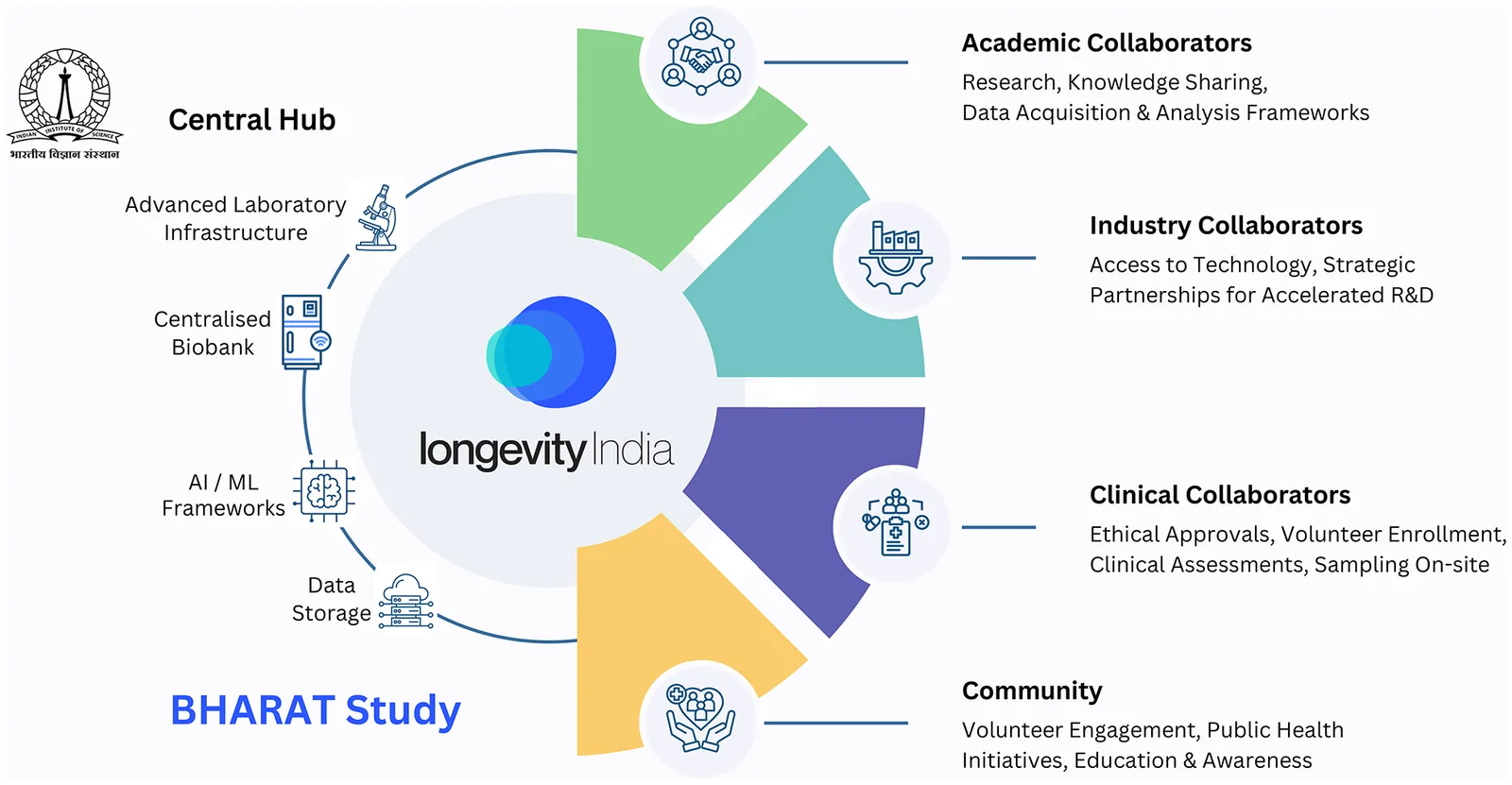
**Study team and collaborative framework of the BHARAT study.** Schematic representation of the hub-and-spoke collaborative model underpinning the BHARAT study, conducted under the Longevity India initiative. The Indian Institute of Science (IISc) functions as the central hub, providing advanced laboratory infrastructure, centralized biobanking, AI/ML and computational frameworks, and secure data storage and governance. Multiple regional clinical partners serve as spokes, facilitating participant recruitment, clinical phenotyping, and the collection of primary data and biospecimens.

### Central hub (IISc)

The Indian Institute of Science serves as the central coordinating hub, hosting infrastructure and expertise for sampling, biobanking, data acquisition, mass-spectrometry based multi-omics data collection, immunophenotyping, development of data analytics and AI/ML pipelines. Dedicated computational infrastructure enables integration of high-resolution molecular data with clinical and lifestyle variables. The hub ensures centralized quality control, protocol standardization, and data governance across participating centres.

### Partner institutions and organisations (Nodes)

The partner hospitals (MS Ramaiah Medical College, Bangalore Baptist Hospital, Sri Madhusudan Sai Institute of Medical Sciences and Research, Bangalore Medical College and Research Institute, and JSS Medical College) serve as regional nodes for participant recruitment and sampling. These include clinics and teaching hospitals equipped with trained medical personnel and infrastructure for screening, standardized sample and metadata collection, temporary storage, and transport of biological samples. Clinical teams are responsible for screening participants, administering questionnaires, conducting physical and cognitive assessments, and ensuring adherence to the inclusion-exclusion criteria. We have collaborated with several companies and communities (Vayah Vikas, Association of Healthcare Providers – India, Dementia India Alliance, Biopeak Wellness) across health and wellness, sports and fitness, senior care, and senior living to ensure diverse representation and recruitment of participants in the study, given that healthy individuals do not visit hospitals. These collaborators serve as additional nodes for identifying and enrolling participants in the study. Other integral nodes in this model include industry partners (TATA 1mg, Lal Path Labs, Decode Age, ARCA AI, Diagnoptics Inc., and others) that provide access to technologies and services, including metagenomics, epigenomics, and diagnostic tests for clinical benchmarking.

### Expertise and governance

The collaborative network comprises clinicians, geriatricians, molecular biologists, engineers, computational biologists, and epidemiologists, supported by industry partners who contribute technological and analytical capabilities. The governance structure follows a centralized coordination model, where uniform operating procedures and data standards are maintained through regular inter-institutional meetings and audits.

The feasibility and scale of the study are enabled by a consortium of over 30 faculty members, 50 clinicians, and 100 trained research and technical staff spanning biology, medicine, and computational sciences. This integrated ecosystem allows seamless coordination between clinical and laboratory arms, ensuring consistency in sample processing, metadata capture, and analytical workflows.

## METHODS

### Study design and setting

Currently, four centres across the state of Karnataka, India are operational, aiming to enrol 5,000 participants (based on approved ethical clearance), with each centre contributing approximately 1,000–2,000 individuals. The design is scalable, allowing expansion to new centres across India under the same standardized framework, with each additional site obtaining independent ethical approval through its community medicine department. This approach ensures both regional representation and long-term adaptability of the cohort.

### Study participants

### Inclusion criteria

Participants in the BHARAT Study are ‘apparently’ healthy volunteers aged 18 years or older, recruited from both hospital-linked and community-based settings. The inclusion criteria are designed to capture a representative cross-section of the healthy Indian population across sex, age, rural–urban strata, and, interestingly, for India, the vegetarian and meat-eating axes. Only individuals free from acute or chronic illnesses are enrolled to establish normative biological and physiological reference ranges across the lifespan.

### Exclusion criteria

Participants with a known history, diagnosis, or clinical evidence of the following conditions are excluded from the study:

- **Chronic cardiac disorders:** Present or past heart failure, cardiomyopathy (any type), or congenital valvular/structural heart disease (with or without treatment).
- **Chronic pulmonary disorders:** Interstitial lung disease, severe chronic obstructive pulmonary disease (GOLD stage ≥3), or pulmonary hypertension of any etiology.
- **Chronic neurological disorders:** Severe cerebrovascular events with residual neurological deficits, recurrent cerebrovascular accidents, neurodegenerative diseases (e.g., dementia, Parkinsonism, motor neuron disease), chronic demyelinating disorders, or progressive neuromuscular diseases (e.g., myasthenia, muscular dystrophy).
- **Chronic gastrointestinal disorders:** Chronic liver disease (≥Stage 2), chronic pancreatitis, or inflammatory bowel disease.
- **Chronic kidney disease:** Stage 3 or higher.
- **Autoimmune disorders:** Any clinically diagnosed systemic or organ-specific autoimmune disease.
- **Transplant recipients:** Individuals with prior solid-organ or bone marrow transplantation.
- **Active or prior malignancy** (solid organ or haematological), including individuals currently undergoing or having completed cancer-directed therapy (chemotherapy, radiotherapy, immunotherapy, or hormonal therapy in the past or surgery in the last 1 year).
- **Recent infections or interventions:** Fever or any active infection within the previous two weeks, or use of antibiotics during that period.
- **Recent alcohol intake:** Consumption of alcohol within one-week preceding enrolment.

### Recruitment and enrollment

Recruitment follows a dual-channel strategy involving (i) hospital-linked screening through participating clinical centres and (ii) community outreach to ensure demographic and geographic diversity. Potential volunteers are informed about the study objectives, procedures, and eligibility requirements prior to screening. Those meeting the inclusion criteria undergo clinical evaluation and questionnaire-based assessments before providing written informed consent (Figure 2).

**Figure 2:**
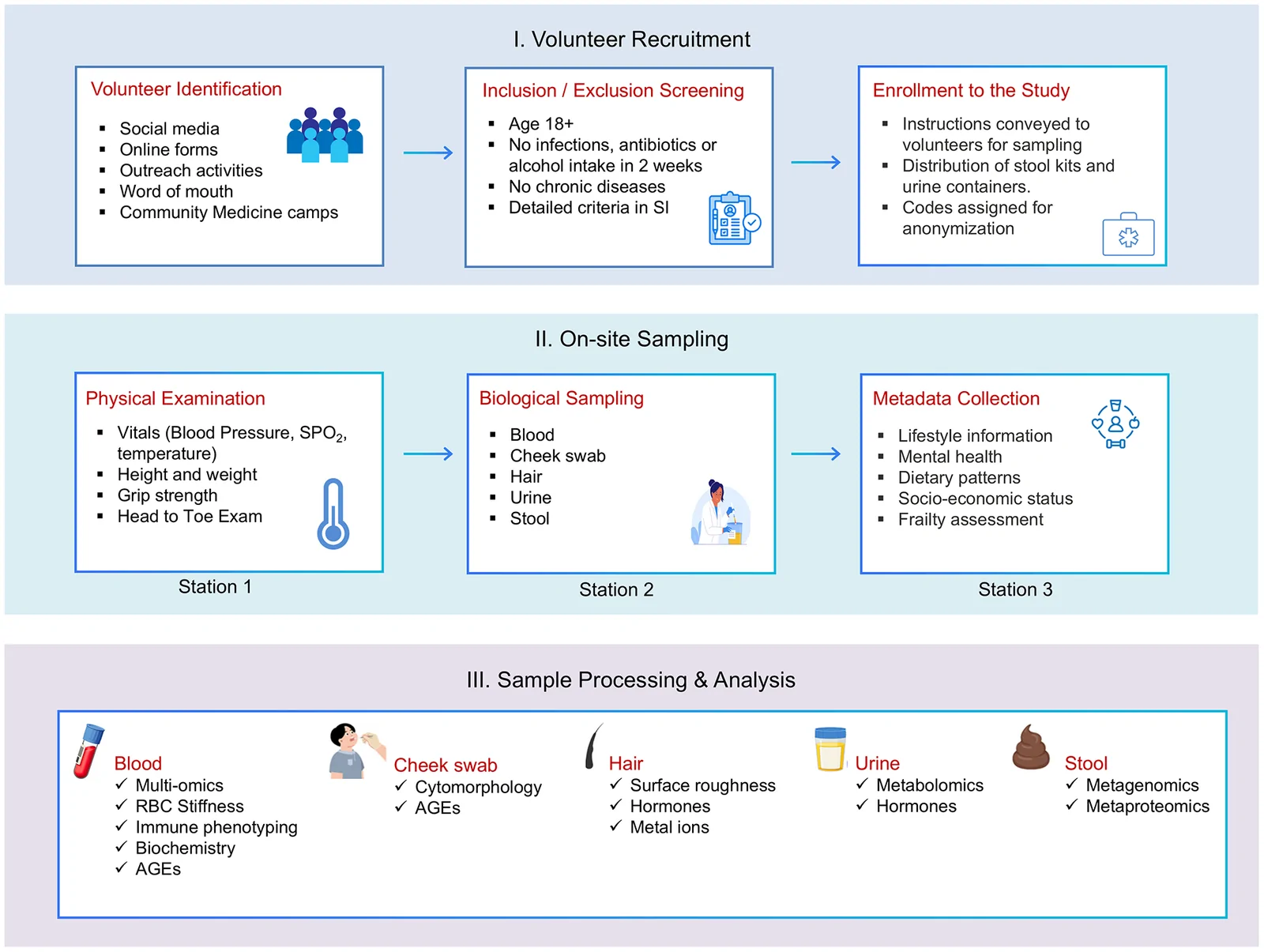
**BHARAT study workflow for recruitment, sampling, and analysis.** Schematic overview of the three-stage study pipeline comprising (I) volunteer recruitment and screening, (II) on-site clinical examination, biospecimen collection, and metadata acquisition, and (III) sample processing and multi-modal analysis. Blood, cheek swab, hair, urine, and stool samples are subjected to complementary molecular, cellular, biochemical, and omics-based assays to enable integrated phenotyping of biological aging across cohorts.

Each eligible participant is assigned a unique identification code enabling secure linkage of clinical, lifestyle, and omics data. This coding system also facilitates the de-identification of data across sites and ensures traceability within the biobanking framework.

### Participant stratification

Participants are stratified into five age groups representing distinct life stages: 18–29, 30–44, 45–59, 60–74, and ≥75 years. Each age stratum maintains balanced representation by sex and rural–urban distribution, capturing India’s demographic diversity. The study design enables both cross-sectional analysis of age-associated biological and physiological variations and future longitudinal extensions through re-sampling and follow-up of select cohorts.

### Metadata collection

All participants undergo a standardized, multi-modal assessment, including structured questionnaires that cover sociodemographic, dietary, physical activity, psychosocial, and quality-of-life information, as well as anthropometric measurements and a clinical vital assessment. The details of the metadata questionnaire are outlined in Supplementary Information.

### Digital infrastructure

All participant data are collected at collaborating hospital and community sites using a secure, custom-designed electronic data capture platform developed in collaboration with ARCA AI. Data entered at participating sites are securely transmitted to the Indian Institute of Science (IISc), which serves as the centralized data repository and institutional custodian for the BHARAT Study. IISc hosts the synchronized database for all clinical, questionnaire, laboratory, and multi-omics data, under institutional oversight and role-based access control. Each participant is assigned a unique anonymized study identifier, enabling deterministic linkage across data modalities while ensuring strict separation of identifiable information from analytical datasets.

### Biological sampling

The following biological specimens are collected from all participants: blood, urine, stool, cheek swab, and hair. All biological specimens are collected under standardized conditions to minimize pre-analytical variability and ensure consistency across clinical sites. Participants fast overnight (8–10 hours), and sampling is conducted the next day between 7:30 and 9:30 a.m. by trained clinical staff, following aseptic procedures.

### Blood

A total of 25 mL of venous blood is drawn using sterile, vacuum-sealed collection tubes. All vials are labelled with pre-assigned barcodes corresponding to participant IDs and immediately logged in the ODK-linked laboratory management system. EDTA-coated tubes are used for blood smears, whole blood, and plasma-based assays. Serum separator tubes (SST) are used for serum-based assays, and Fluoride-coated vials for blood biochemical assays. After collection, the EDTA tubes are gently inverted to prevent clotting. Peripheral blood smears for cytochemistry analysis are prepared and fixed with 100% methanol within 45 minutes of collection. Plasma separation is performed by centrifugation at 1200 × g for 10 minutes, followed by plasma extraction, aliquoting, and snap-freezing in liquid nitrogen. SST tubes are kept upright at room temperature to facilitate blood clotting for 10 minutes, followed by centrifugation at 1200 × g for 10 minutes. Serum is separated, aliquoted, and stored at 4° C for analysis or snap-frozen in liquid nitrogen for biobanking. Blood biochemistry is performed on fresh samples at a partner diagnostic laboratory. The complete list of tests is included in Supplementary Information.

### Urine

Participants are provided with sterile urine collection containers and 5 mL cryovials. Midstream urine is collected in the morning, transferred aseptically into cryovials, snap-frozen in liquid nitrogen, and transported for biobanking.

### Stool

Participants are given a stool collection kit (Decode Age) containing sterile containers with DNA stabilizer, spatulas, gloves, and illustrated instructions. Samples are collected within 24 hours of the sampling visit and stored temporarily at room temperature. The samples are collected the following day, transported and the aliquots are transferred to cryovials for storage at –20° C.

### Cheek swab

Buccal epithelial cells are obtained using sterile cotton swabs. Each participant rinsed the mouth before collection. The swab is rotated along the inner cheek surface for 30 seconds on each side. Cells are transferred onto a marked region on a glass slide using a PAP pen, fixed in 10% neutral buffered formalin (NBF) for 45 minutes, and stored in phosphate-buffered saline (PBS) at 4° C until processing.

### Hair

Approximately 10 strands of scalp hair are cut 0.5 cm close to the scalp from the posterior vertex using sterile scissors. The proximal 3 cm of each strand is retained, representing approximately three months of growth. Hair samples are stored at room temperature in a desiccated condition for subsequent analysis.

### Transport and storage

All samples are transported to the central biobank at IISc using temperature-controlled containers (cool boxes with ice packs or liquid nitrogen dewars). Chain-of-custody logs are maintained for each sample batch. At IISc, samples are organized by specimen type and transferred into long-term storage under the following conditions:

- Blood, serum, plasma and urine samples are stored at –150° C.
- Cheek swabs and blood smears are stored at 4° C
- Hair samples are stored at ambient temperature.

Each sample is assigned a unique digital code linking metadata, processing status, and future analytical use within the Longevity India biobank infrastructure.

### Sample processing and multi-omics analysis

Following receipt at the IISc central biobanking facility, samples are processed within a controlled biospecimen handling pipeline in accordance with the BHARAT Study SOPs. The objective is to generate integrative molecular datasets spanning genetic, epigenetic, proteomic, metabolomic, lipidomic, immunophenotypic, and microbiome dimensions. Our multi-omics strategy integrates:

*Epigenomics:* DNA methylation profiling using Infinium Methylation Screening Array (270,000 CpG sites) and SNP genotyping using Axiom Asia Precision Medicine Array (750,000 markers tailored for Southeast Asian populations).

*Proteomics:* Untargeted plasma proteomics via nano-liquid chromatography-tandem mass spectrometry (nLC-MS/MS (EvoSEP Eno), Bruker timsTOF HT platform), complemented by targeted protein/peptide panels and cytokine panels (aptamer-based platforms).

*Metabolomics and Lipidomics:* Untargeted plasma and urine metabolomics via high-resolution LC-MS (Agilent UHPLC coupled to Bruker timsMetabo), employing modified Bligh-Dyer and Matyash extraction protocols.

*Metagenomics:* Shotgun metagenomic sequencing of stool samples via Oxford Nanopore Technology (ONT) platform for species- and function-level characterization of gut microbial communities.

*Immune Phenotyping:* Deep immunophenotyping using Beckman Coulter DuraClone™ dried antibody panels (T cell, B cell, granulocyte panels) via flow cytometry, assessing naïve, memory, senescent, and activated immune subsets.

*Cellular Aging Markers:* Advanced glycation end-product (AGE) quantification via immunocytochemistry on peripheral blood smears and buccal cells, red blood cell stiffness assessment via microfluidic devices, and hair surface roughness analysis via optical imaging.

Detailed protocols of sample processing and data collection are available in the Supplementary Information section.

### Data management and integration

All multi-modal and multi-omics data are managed and stored at IISc, which maintains the common repository. Data architecture includes a local server (master node) with role-based access and routine backups, adhering to both HIPAA standards and Indian Data Protection and Privacy laws. We track samples using in-house laboratory information management software with data audit trails and instrument API integrations. We integrate all datasets (clinical metadata, laboratory measurements, multi-omics data) by linking each unique anonymized participant identifier to specimen and assay-level identifiers. We perform preprocessing, normalization, and multi-omics integration using standardized pipelines before downstream modelling of biological age, resilience, metabolic indices, and frailty using machine learning frameworks.

## DISCUSSION

The BHARAT Study represents India’s first large-scale, multi-omics investigation into biological aging and health trajectories, addressing a critical gap in global aging research. While landmark cohorts such as the UK Biobank (500,000 participants, predominantly European ancestry) [60], the All of Us Research Program (245,388 participants, 77% from underrepresented populations but primarily US-based) [61], and the Framingham Heart Study [62] have profoundly shaped our understanding of aging biology, Asian populations, which represent nearly 60% of the global population, remain conspicuously underrepresented within such studies (Figure 3). Among the limited Asian cohorts, the Singapore-based ABIOS study (420 participants, aged 21 and above, cross-sectional study design with epigenomics, proteomics, and metabolomics) [63] provides the closest methodological parallel to the BHARAT cohort, albeit at a substantially smaller scale (Figure 4). The Japanese Tohoku Medical Megabank (encompassing 150,000 participants with genomics and metabolomics) primarily focuses on disaster-affected populations and their health outcomes, although its tertiary assessments from 2021 have now begun to examine aging trajectories [64].

**Figure 3:**
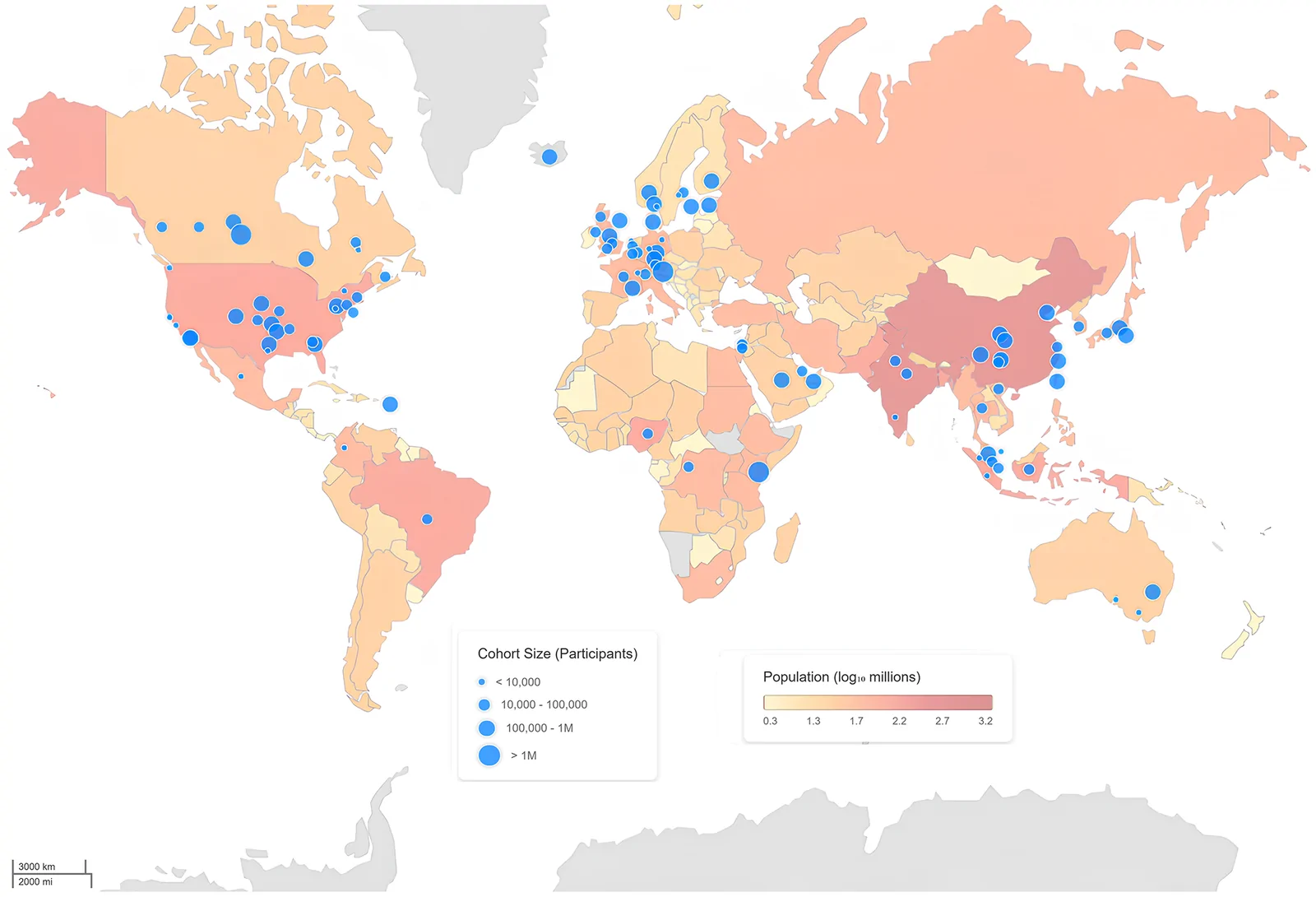
**Global distribution of multi-omics cohorts studying healthy populations.** World map depicting the geographic distribution of multi-omics studies conducted in healthy volunteers. Blue circles indicate the location of individual cohorts, with circle size proportional to cohort size (number of participants). Country-level population size (log scale, millions) is overlaid as a choropleth to provide demographic context. The map highlights the global concentration and regional disparities of existing multi-omics cohorts generating at least one omics dataset from biological samples (See Supplementary Information for details of datasets and analysis).

**Figure 4:**
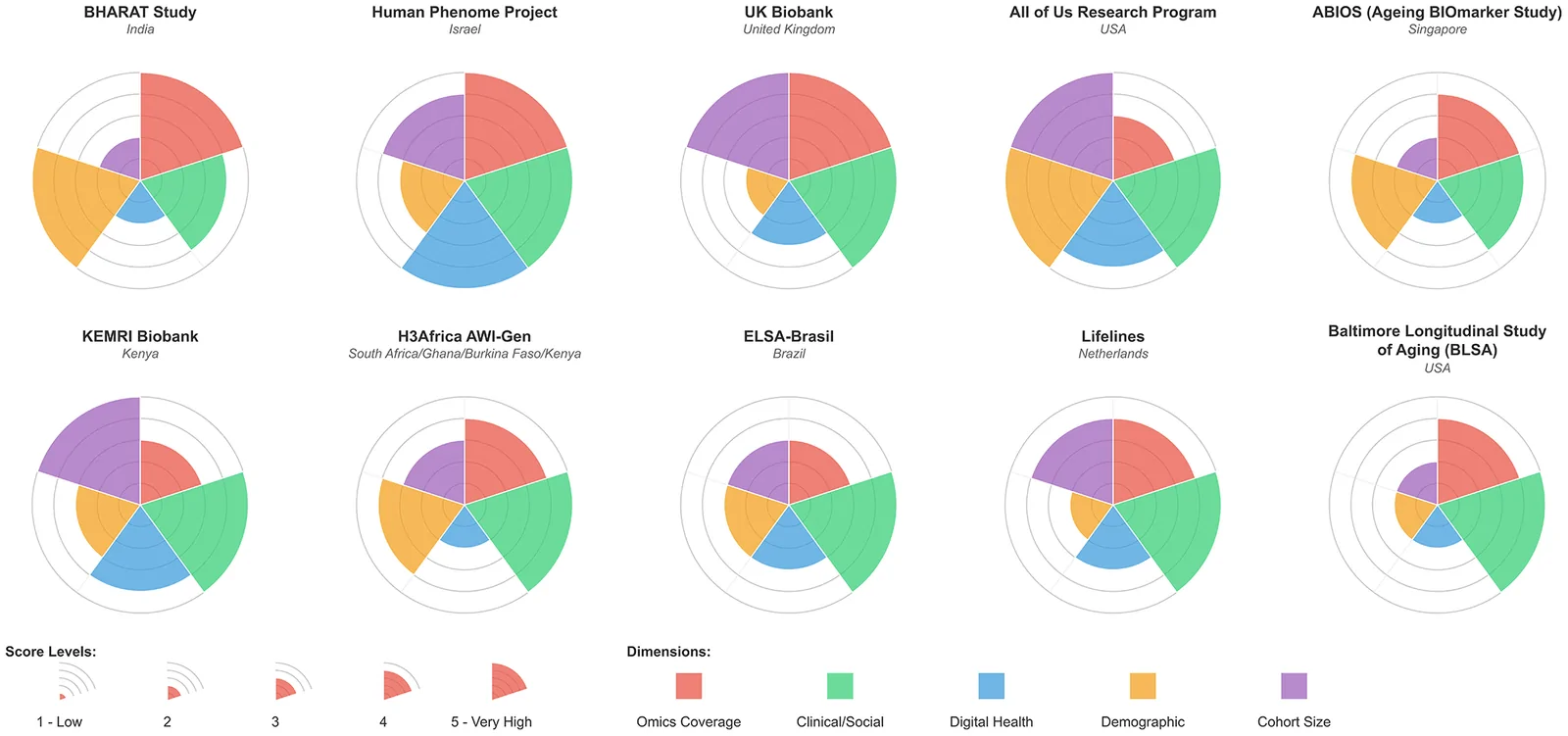
**Radar plots of representative worldwide multi-omics cohorts scored on 5 different criteria: Omics coverage, clinical/social metadata collection, use of digital health tools, demographic coverage, and cohort size.** Each category is scored on a scale of 1-5. The in-depth criteria and justification for scoring are given in Supplementary Information.

Despite these advances, the datasets that train most aging clocks, molecular risk predictors, and longevity biomarkers are derived almost exclusively from Western populations, limiting their applicability in Asian contexts. Population-level differences in genetic ancestry, infection burden, nutrition, environmental exposures, socioeconomic backgrounds and healthcare access have a significant influence on biological aging signatures [65–68]. The absence of Indian representation in such datasets, therefore, constrains both the calibration and interpretive power of aging models. There is a pressing need to establish population-specific, multi-dimensional datasets that reflect the unique biological and socio-environmental heterogeneity of India. The BHARAT Study aims to directly address this gap by establishing an integrated, multi-omics study cohort of Indian adults across diverse geographic, cultural, and socio-economic backgrounds. Its sampling framework is geared to balance representation across sex, age, income, and rural-urban strata, thereby creating a robust reference landscape for understanding variability in physiological and molecular parameters. Current clinical benchmarks in India are largely extrapolated from Western reference intervals, with few longitudinal or cross-sectional datasets defining normative ranges for healthy Indian adults, such as those from Phenome India [69]. By systematically characterizing healthy individuals across strata, this study provides the first dataset to recalibrate biomarker thresholds, reference intervals, and health trajectories specifically within the Indian context. Longitudinal studies in health trajectories and disease burden assessment have been done in India by two major focused studies, The Longitudinal Ageing Study in India (LASI) [70] and Indian Study of Healthy Ageing (ISHA) [71]. Both studies collect high-quality anthropomorphic data and basic molecular features but lack in-depth untargeted omics profiling, which is the unique aspect of the BHARAT.

This study builds upon methodological foundations established by pioneering aging cohorts while addressing critical gaps in population representation. The Stanford 1000 Immunomes Project (n=1,001, ages 8-96, 12-year longitudinal follow-up), demonstrated that deep immune phenotyping combined with machine learning can identify inflammatory biomarkers, most notably the iAge clock centered on CXCL9 that predicts multimorbidity, frailty, and cardiovascular aging independent of chronological age [46]. This work established inflammaging as a quantifiable, mechanistically actionable hallmark of biological aging and pioneered the use of deep autoencoders for high-dimensional immunological data, methods we aim to adapt for our own multi-omics integration. The InCHIANTI study (Italy, n=1,200, ages 65+) provided proteomic and metabolomic aging signatures validating that circulating biomarkers track with physical function, frailty, and mortality across European populations [72]. In India, the Council of Scientific and Industrial Research (CSIR) Phenome India initiative announced a large-scale multi-omics cohort across 10,000 CSIR employees, pensioners, and their spouses for cardiometabolic disease risk prediction from diverse geographic regions [69]. Phenome India exclusively recruits CSIR employees and their families, a relatively homogenous, urban, educated cohort with stable employment, limiting generalizability to India’s broader socioeconomic and rural-urban diversity. BHARAT complements this effort by targeting community-based recruitment spanning rural and urban sites across diverse socioeconomic strata, with explicit focus on aging biology and developing population-specific molecular aging features validated against health trajectories.

A distinguishing strength of the BHARAT study lies in its multi-omics multi-feature data collection design. While pioneering global aging cohorts such as the Stanford 1000 Immunomes Project, Baltimore Longitudinal Study of Aging [73], and Multi-Ethnic Study of Atherosclerosis [74] established foundational frameworks for immune aging, biological clocks, and phenotypic trajectories, they predominantly employed targeted proteomic platforms such as Olink or SomaLogic. These aptamer-based approaches, though highly reproducible and scalable, are limited to pre-selected analytes and cannot discover novel biomarkers outside their fixed panels [63, 75]. BHARAT incorporates an unbiased, discovery-driven LC-MS/MS pipeline for proteomics, metabolomics, lipidomics, and molecular profiling, enabling hypothesis-free detection of novel metabolites, post-translational modifications, and population-specific signatures absent from Western reference databases. These layers are complemented by targeted panels for hormones, metabolites, cytokines, and clinical biochemistry, creating a comprehensive, clinically relevant, and usable biochemical framework for integrative analyses. Further, deep immune phenotyping adds granularity to cellular and functional heterogeneity within the Indian context, a critical aspect in a tropical country with high exposure to infectious diseases and chronic inflammatory states [76]. Together, these datasets enable the identification of systemic, multi-modal biomarkers that capture health, resilience, transitions through various health phases in life and quality of health decline across the aging spectrum.

Furthermore, the microbiome analysis included in the study extends beyond conventional 16S rRNA-based approaches and employs nanopore-based whole-metagenome shotgun sequencing, enabling species- and function-level characterization of microbial communities and their derived metabolites. Given India’s extraordinary dietary diversity, fermentation practices, infectious disease burden and sanitation gradients, such data are important for decoding microbiome-mediated influences on metabolic and immune aging. Standardized protocols for sample collection, stabilization, and sequencing depth have been optimized for reproducibility, ensuring technical comparability with other global cohorts while retaining contextual specificity.

In addition to deep molecular profiling described above, the study’s metadata and phenotypic framework provide a rich contextual foundation for multi-omics interpretation. Comprehensive data on clinical history, comorbidities, anthropometry, diet, sleep, physical activity, mental health (DASS-21), cognitive performance (MMSE), quality of life (WHO-QOL) and frailty are systematically collected through standardized instruments and approaches by the clinical teams. These have been translated and culturally adapted into regional languages to ensure conceptual equivalence and reduce respondent bias.

Collectively, the BHARAT Study is positioned to deliver multiple scientific outputs. These include (i) validated biomarkers and reference datasets for healthy aging in Indian adults, (ii) integrative pipelines for multi-omics data analysis and biological age or physiologically relevant wellness state estimation, (iii) retraining and calibration of biological clocks for non-Western populations, and (iv) generation of high-quality data for machine learning models and diagnostic development. In the broader context of global geroscience, this study establishes a scientifically rigorous framework for understanding biological aging in a highly diverse population. By generating interoperable, high-resolution data suited for mechanistic modelling and machine learning, BHARAT contributes a resource of global relevance that would be capable of refining universal models of aging biology while revealing novel, population-specific pathways that inform prevention and intervention strategies**.** Integrating these models with clinical and lifestyle data will facilitate the development of personalized risk and overall mortality prediction tools and diagnostic panels tailored to Indian populations.

Beyond biomarker discovery, BHARAT’s cross-layered datasets can uncover mechanistic nodes that link metabolic, immune, and microbial alterations to aging phenotypes, unlocking targets for therapeutic interventions. These insights can qualify and enrich the design of dietary, pharmacological, and lifestyle interventions aimed at promoting healthy aging. In parallel, the study’s biobanking infrastructure and standardized analytical pipelines will enable partnerships with national and international consortia, ensuring that BHARAT contributes both to global knowledge and to the development of contextually relevant strategies for extending healthspan.

## Supplementary Material

Supplementary Information

Supplementary Contributors Information
