# Supplementary material for: The BHARAT study: a multi-modal, multi-omics investigation of aging signatures in the Indian population
Source: Aging (Albany NY). 2026 Apr 24;18(1):380–96. doi: 10.18632/aging.206373 (PMC13632655; doi:10.18632/aging.206373)
Supplement: Supplementary file 1 [file aging-18-1-206373-s001.docx]

**Supplementary Information**

**The BHARAT Study: A Multi-Modal, Multi-Omics Investigation of Aging Signatures in the Indian Population**

Suramya Asthana^1,2^, Amruth Deepak Bhat^1,3^, Gayathri Mahadevan^1,2^, Lakshmi Kothegala^1,2^, Swati Negi^1,2^, Seema Yadav^1,2^, Anupama Sudhakaran^1,2^, Annwesha Roy^1,2^, Meet Makwana^1,2^, Rahul Patel^1,2^, Harshavardhan Rao B^4^, Caroline Elizabeth George^5^, Srimonta Gayen^1,2^, Shantanu Shukla^1,2^, Siddharth Jhunjhunwala^1,3^, Prosenjit Sen^1,6^, Prabhdeep Kaur^1,7^, Ramray Bhat^1,2,3^, Deepak Kumar Saini^1,2,3^* on behalf of the Longevity India Team^#^ and Collaborators

**Corresponding Author-* [*deepaksaini@iisc.ac.in*](mailto:deepaksaini@iisc.ac.in)

^#^Complete list of contributors (not listed above) is provided below.

**Author Affiliations:**

^1^Longevity India, Division of Interdisciplinary Sciences, Indian Institute of Science, Bengaluru 560012

^2^Department of Developmental Biology and Genetics, Division of Biological Sciences, Indian Institute of Science, Bengaluru 560012

^3^Department of Bioengineering, Division of Interdisciplinary Sciences, Indian Institute of Science, Bengaluru 560012

^4^Department of Gastroenterology, MS Ramaiah Medical College, RUAS, Bengaluru 560054

^5^Community Health, Palliative Care & Research Division, Bangalore Baptist Hospital, Bengaluru 560032

^6^Centre for Nano Science and Engineering, Division of Interdisciplinary Sciences, Indian Institute of Science, Bengaluru 560012

^7^Isaac Centre for Public Health, Tata IISc Medical School, Indian Institute of Science, Bengaluru 560012

**Study Contributors**

| **Name** | **Affiliation** |
| --- | --- |
| ***^#^Longevity India Team*** | |
| Ambarish Ghosh | ^1^Longevity India, Division of Interdisciplinary Sciences, Indian Institute of Science, Bengaluru 560012  ^6^Centre for Nano Science and Engineering, Division of Interdisciplinary Sciences, Indian Institute of Science, Bengaluru 560012  ^8^Department of Physics, Indian Institute of Science, Bangalore 560012, India |
| Ananya Sarkar, Anchita Gopikrishnan, Annapoorni Rangarajan, Ayushi Verma, Inbasekar C, Jalak Mehta, Jayant Sehgal, Nikhil R Gandasi, Sagarika Prabhakar, Shweta Thakar, Yogesh | ^1^Longevity India, Division of Interdisciplinary Sciences, Indian Institute of Science, Bengaluru 560012  ^2^Department of Developmental Biology and Genetics, Division of Biological Sciences, Indian Institute of Science, Bengaluru 560012 |
| Anuva R, Bishal Singh, Nischitha Baliga, Shovamayee Maharana, Subba Rao GS, Vinod Bhat | ^1^Longevity India, Division of Interdisciplinary Sciences, Indian Institute of Science, Bengaluru 560012  ^9^Department of Microbiology and Cell Biology, Division of Biological Sciences, Indian Institute of Science, Bengaluru 560012 |
| Kaushik Chatterjee | ^1^Longevity India, Division of Interdisciplinary Sciences, Indian Institute of Science, Bengaluru 560012  ^3^Department of Bioengineering, Division of Interdisciplinary Sciences, Indian Institute of Science, Bengaluru 560012  ^10^Department of Materials Engineering, Indian Institute of Science, Bengaluru 560012 |
| Mohit Kumar Jolly | ^1^Longevity India, Division of Interdisciplinary Sciences, Indian Institute of Science, Bengaluru 560012  ^3^Department of Bioengineering, Division of Interdisciplinary Sciences, Indian Institute of Science, Bengaluru 560012 |
| Narendra Dixit | ^1^Longevity India, Division of Interdisciplinary Sciences, Indian Institute of Science, Bengaluru 560012  ^3^Department of Bioengineering, Division of Interdisciplinary Sciences, Indian Institute of Science, Bengaluru 560012  ^11^Department of Chemical Engineering, Indian Institute of Science, Bengaluru 560012 |
| Swaminathan S | ^1^Longevity India, Division of Interdisciplinary Sciences, Indian Institute of Science, Bengaluru 560012  ^3^Department of Bioengineering, Division of Interdisciplinary Sciences, Indian Institute of Science, Bengaluru 560012  ^12^Department of Nephrology, Tata IISc Medical School, Indian Institute of Science, Bengaluru |
| Sanjiv Sambandan | ^1^Longevity India, Division of Interdisciplinary Sciences, Indian Institute of Science, Bengaluru 560012  ^13^Instrumentation and Applied Physics, Indian Institute of Science, Bengaluru 560012 |
| Chiranjib Bhattacharya | ^1^Longevity India, Division of Interdisciplinary Sciences, Indian Institute of Science, Bengaluru 560012  ^14^Department of Computer Science and Automation, Indian Institute of Science, Bengaluru 560012 |
| Arvind Ramanathan | ^15^Institute for Stem Cell Science and Regenerative Medicine (InStem), Bangalore Life Science Cluster, Bengaluru 560065 |
| Sanjeeva Srivastava | ^16^Department of Biosciences and Bioengineering, Indian Institute of Technology Bombay, Mumbai 400076 |
| Tavpritesh Sethi | ^17^Computational Biology, Indraprastha Institute of Information Technology, Delhi 110020 |
| Vishal Rao US | ^18^Department of Head and Neck Oncology, HCG Cancer Center, Bengaluru 560027 |
| ***Clinical Partners*** | |
| Maddipati Tatarao, Suman Sarah Varghese, Manjula KS, S Praveena, Sharon Rimai | ^5^Community Health, Palliative Care & Research Division, Bangalore Baptist Hospital, Bengaluru 560032 |
| Shreya Thadigotla, Prateek Reddy, Drashti Godhani, Anagha Pritvi Raj, Bhoomika Y, Harshit V, | ^4^Department of Gastroenterology, MS Ramaiah Medical College, RUAS, Bengaluru 560054 |
| Sathish Babu K.N., Phaneendra Mallekavu | ^19^Sri Madhusudan Sai Institute of Medical Sciences and Research, Chikkaballapur 562103 |
| Anil S. Bilimale, Sheeba B, Divya K L, Hemanth R, Aishwarya N | ^20^School of Public Health, JSS Medical College, JSS AHER, Mysuru 570015 |
| Shreya Gudi, Raghvendra BM | ^21^Department of Geriatric Medicine, Bangalore Medical College & Research Institute, Bengaluru 560002 |
| Amit Bahuguna, D Navya, Devyani Sapra, Ashwin P Nair, Vishal Gautam | ^22^Command Hospital, Bengaluru 560007 |
| ***Industry Partners*** | |
| Jagan L, Aditi Muglikar, Darshit Patel | ^23^Centenarians Life Sciences Pvt. Ltd. (Decode Age) |
| John Mulder | ^24^Diagnoptics Technologies b.v. |
| Tushar Asthana, Shibu V Ignatious, Ramya Satish Kumar, Karthik Iyer, Rohit Rajan | ^25^ARCA AI |
| Arpita Roy Dam, Prashant Nag, Vinisha Nahata, Swati Gupta, Shanmugam RK, Gaurav Agarwal | ^26^TATA 1mg Labs |
| Alok Sharma, Vandana Lal, Archana Grover | ^27^Lal Path Labs |

***Metadata Questionnaires.*** Data acquisition follows a modular framework consisting of standardized and validated instruments, designed to capture multidimensional determinants of biological aging. The comprehensive BHARAT Study Questionnaire comprises the following sections:

1. **Sociodemographic Profile** – Age, sex, education, occupation, income, marital status, religion, primary language, and geographic indicators (sampling location).
2. **Lifestyle and Environmental Factors** – Living arrangements, residential setting (urban/rural), and exposure to passive smoke or environmental pollutants.
3. **Dietary Habits and Nutrition** –
   - *Dietary pattern*: meal frequency, vegetarian/non-vegetarian status, frequency of breakfast and fast-food consumption.
   - *Food frequency list*: standardized intake frequency (daily, weekly, monthly, occasional, or never) across major food groups – cereals, pulses, vegetables, fruits, nuts, animal products, sugars, fats, and processed foods.
   - *Probiotic and supplement use*: frequency, type, and reason for consumption (e.g., immunity, digestive health).
4. **Physical Activity and Sedentary Behaviour** – Frequency and duration of low-, moderate-, and high-intensity physical activities (walking, cycling, household work, or exercise).
5. **Substance Use and Addiction History** – Smoking (cigarette, bidi, hookah, e-cigarette) with *Heaviness of Smoking Index*; smokeless tobacco (type, frequency, duration); alcohol consumption (frequency, quantity, CAGE screening); and other substance use.
6. **Sleep and Circadian Health** – Sleep duration, latency, nocturnal awakenings, and self-reported sleep satisfaction.
7. **Cognition and Functional Health** – *Mini-Mental State Examination (MMSE)* to assess cognitive performance, *FRAIL Scale* to evaluate frailty status, and *WHO-QOL-BREF* for quality-of-life domains.
8. **Personal and Family Medical History** – Self-reported history of hypertension, diabetes, hypothyroidism, asthma, ischemic heart disease, or psychiatric illness; COVID-19 infection or vaccination history; and family history of metabolic, cardiovascular, renal, or neurodegenerative diseases.

***Clinical and Physical Examination.*** All participants undergo a standardized clinical examination performed by trained medical personnel at the partner hospital sites. Parameters recorded include:

- **Vital Signs:** Pulse rate, blood pressure (systolic/diastolic), respiratory rate, oxygen saturation, and body temperature.
- **Anthropometry:** Height, weight, and calculated body mass index (BMI).
- **General Physical Examination:** Pallor, icterus, cyanosis, clubbing, lymphadenopathy, pedal edema, and thyroid examination.
- **Systemic Examination:** Cardiovascular, respiratory, gastrointestinal, and musculoskeletal systems assessed for normality or abnormalities.
- **Functional and Biophysical Tests:**
  - *Hand-grip strength* (using calibrated dynamometer) as a marker of musculoskeletal performance.
  - *Single-breath counting test* for respiratory reserve.
  - *AGE Reader* for non-invasive measurement of skin advanced glycation end-products.

All measurements are logged directly into the digital platform to minimize transcriptional error. Quality control measures include periodic calibration of devices and inter-observer standardization across clinical centres.

***Blood Biochemistry.*** The blood biochemistry panel includes the following tests - Complete Blood Count, Liver Function Test, Kidney Function Test, Fasting Blood Sugar, HbA1c (Glycosylated Hemoglobin), Insulin Fasting, Lipid profile, Apolipoprotein – A1, Thyroid Profile Free, 8AM Cortisol, Homocysteine, Vitamin D (25-OH), Iron Serum, Total Iron Binding Capacity, Ferritin, Vitamin B12, Vitamin B9 (Folic Acid), C-Reactive Protein Quantitative and Erythrocyte Sedimentation Rate.

***Epigenomics:*** The study will use the Infinium Methylation Screening Array (MSA, Illumina) for high-throughput genome-wide methylation profiling. MSA encompasses 270,000 unique methylation sites linked to human traits and diseases, including neurodegenerative, cardiovascular, respiratory, and renal disorders. It also covers loci related to development, aging, ancestry, autoimmune diseases, and rare genetic disorders [1]. We will follow Illumina EX methylation assay protocol. Genomic DNA will be isolated from a frozen whole blood sample using the QIAamp Blood DNA Mini kit (Cat #51106), and its quality will be assessed using a nanodrop spectrophotometer (Thermo Scientific) and a Qubit 4 fluorometer (ThermoFisher Scientific). Approximately 500ng of genomic DNA from each sample will be used for Zymo EZ DNA Methylation Lightning MagPrep Kit for bisulfite conversion, in which unmethylated cytosine gets converted to uracil, and methylated cytosines are protected from conversion due to the presence of methyl groups.

Following bisulfite conversion, the DNA will be amplified, fragmented, and hybridized to the bead chip, which will then be stained and imaged. Raw IDAT files will be processed using the SeSAMe R package, which is used for pre-processing, p-value-based filtering, normalization, and beta value generation [2]. The beta value, which is a measure of the relative degree of methylation at a locus, is used for downstream analysis and interpretation. Differential methylation analysis will be performed between different age groups to identify DNA methylation-based biomarkers of aging. A comparative analysis of biological age prediction between the available blood-based DNA methylation clocks: Horvath [3], Hannum [4], GrimAge [5], PhenoAge [6], Dunedin PACE [7] will be done to identify the best age predictor for the Indian population.

***SNP Genotyping:*** Single nucleotide polymorphism (SNP) is the variation of nucleotides at similar position in DNA among individuals. SNP can change encoded amino acids, have effect on gene expression, phenotypic traits and is responsible for diversity in a population. In the BHARAT study, Axiom Asia Precision Medicine Array (APMRA) will be used for high-throughput profiling of genetic variants in five age groups across the Indian population. This array is tailored for the Southeast Asian population, comprising 750,000 markers that include loss-of-function, clinical, and disease risk-associated variants [8]. This will help elucidate several genetic pathways that influence aging processes, metabolism, and the risk of age-related pathologies. This data will help in the development of new SNP-based markers for fast and accurate diagnosis of age-related debilitating diseases. For this analysis, we will be using genomic DNA isolated from frozen blood using the QIAamp Blood DNA Mini kit (Cat #51106). Raw CEL files will go through pre-processing and quality check to remove low quality SNPs. Then, for the downstream GWAS analysis variant calling files (VCF) will be used [9]. We will do functional annotation of genetic variants to investigate their association with biological pathways of aging. We can also identify age associated Methylation quantitative trait loci (meQTL) where SNPs are associated with variation in DNA methylation at certain CpG sites [10].

***Immune Phenotyping*:** Immunophenotyping uses Beckman Coulter DuraClone™ dried antibody panels to ensure standardization and reproducibility. Whole blood (0.6 mL) is collected in EDTA tubes and processed either within 2–4 hours or stabilized for transport using a fixation protocol. Three panels are employed: a T cell panel (CD3, CD4, CD8, CD27, CD28, CD45, CD45RA, CD57, CCR7, PD-1), a B cell panel (CD19, CD21, CD24, CD27, CD38, CD45, IgM, IgD), and a customized granulocyte panel (CD15, CD10, CD16, CD33, CD11b, CD11c, PD-L1, lineage cocktail, CD62L, CD45). Together, these allow detailed assessment of naïve, memory, senescent, and activated subsets relevant to aging biology.

For each sample, 100 µL of whole blood is added directly into the DuraClone tubes, and the tubes are vortexed briefly to ensure antibody reconstitution, followed by incubation for 15–20 minutes at room temperature in the dark. Red blood cells are then lysed using VersaLyse Lysing Solution (Beckman Coulter, Cat. No. A09777), added at 2 mL per tube, and incubated for 15 minutes at room temperature. The samples are washed once with 2 mL of phosphate-buffered saline (PBS, room temperature), centrifuged, and resuspended in PBS prior to acquisition. Data are acquired on a DxFLEX/CytoFLEX flow cytometer with a target of 100,000 leukocyte events per sample. Daily cytometer quality control and regular compensation setup are performed to minimize technical variability.

A modified version of the above protocol will be used for sampling when time window is broader, which involves the use of a fixative to enable transport of samples from locations that are far away from the central site (IISc). In this protocol, the only change would be the use of IOTest 3 Fixative Solution (Beckman Coulter, Cat. No. A07800) along with VersaLyse lysing buffer (Beckman Coulter, Cat. No. A09777), followed by refrigerated transport (2-8⁰C) to preserve staining quality, and acquisition on the flow cytometer within 48 h of fixation.

***Red Blood Cell and Cheek Cell Cytochemistry:*** Immunocytochemistry is performed on peripheral blood smears and buccal (cheek) cells to detect the advanced glycation end product (AGE) - *Nε*-carboxymethyl-lysine (CML) following standardized laboratory procedures. Slides with fixed cells are permeabilized with 0.5% Triton X-100 (HiMedia, MB031-100ML), blocked with 3% bovine serum albumin (BSA; SRL, 85171) in 0.1% Triton X-100, and incubated overnight at 4°C with an anti-CML primary antibody (Abcam, ab125145) diluted 1:300. After washing, slides are incubated with Alexa Fluor 488-conjugated anti-mouse secondary antibody (Invitrogen, A-11008; 1:700) for 2 hours at room temperature, followed by DAPI counterstaining (Thermo Fisher Scientific, D1306) for buccal cells. Mounted slides are imaged using an Olympus IX73 semi-confocal microscope under constant acquisition settings to ensure comparability across samples. Image analysis is performed using Fiji (ImageJ) software to quantify fluorescence intensity and assess staining distribution.

***Red Blood Cell stiffness:*** Red blood cell (RBC) deformability is assessed using a polydimethylsiloxane (PDMS)-based microfluidic device designed to simulate microcapillary flow. PDMS base and curing agent are mixed in a 10:1 ratio, thoroughly blended, degassed under vacuum for 2 hours, and cured on a silicon master mold containing the device design at 100°C for 2 hours. The cured PDMS layer is demolded, inlet and outlet ports are punched, and the device is bonded to a glass slide following plasma treatment to activate hydrophilicity. The bonded chips are baked at 60°C for 30 minutes to strengthen adhesion and stored in a dust-free environment until use.

For each experiment, approximately 200 µL of anticoagulated whole blood is centrifuged at 3500 rpm for 10 minutes, and the supernatant is discarded. About 65 µL of the RBC pellet is resuspended in 3 mL of 1× PBS. The suspension is allowed to settle briefly to remove aggregates and then gently resuspended before loading into the device. The device is mounted on an inverted microscope equipped with a 20× objective. Inlet and outlet tubing are connected carefully to prevent air bubble formation. A syringe pump maintains a constant flow rate of 40 mL/h, driving the RBC suspension through the PDMS microchannels. Real-time imaging is performed using a high-speed camera at 1000 frames per second, and three 11-minute recordings are acquired per sample. Video data are processed and analyzed using PFV 4×64 software to extract parameters such as cell transit velocity and deformation behavior, enabling quantitative evaluation of age-related variations in RBC stiffness and flexibility.

***Plasma Proteomics*:** Untargeted proteomic analysis will be performed on snap-frozen plasma samples. Upon thawing on ice, samples will be clarified by centrifugation to remove lipids and cellular debris. Total protein concentration will be determined using a BCA assay. A standardized amount of protein from each sample will be subjected to acetone precipitation at -20°C to concentrate the proteins and remove interfering substances. The resulting protein pellets will be resuspended, reduced with dithiothreitol (DTT), and alkylated with iodoacetamide (IAA) to irreversibly denature the proteins. Proteolysis will subsequently be carried out overnight at 37°C using sequencing-grade trypsin. The resulting peptide mixtures will be acidified and desalted using C18 solid-phase extraction. Purified peptides will then be analyzed by nano-liquid chromatography-tandem mass spectrometry (nLC-MS/MS) using gradients and methods optimized in collaboration with Bruker Daltonics for the timsTOF HT platform.

***Plasma Metabolomics & Lipidomics:*** Lipids and metabolites will be extracted using a modified Bligh and Dyer biphasic protocol followed by an adapted Matyash method to enhance recovery of both polar and non-polar metabolites [11, 12]. Plasma will be centrifuged, and the supernatant will be incubated at –80 °C overnight for protein precipitation. The clarified supernatant will be divided for lipid and metabolite extraction. For lipids, a methanol:chloroform (2:1, v/v) mixture will be added for biphasic separation, followed by centrifugation. The lower organic phase will then be vacuum-dried and stored. For metabolites, methanol (1:4, v/v) will be added to the remaining supernatant, vortexed, and stored at –80 °C for overnight. Samples will then be centrifuged (14,000 × g, 10 min), and the metabolite-rich phase will be dried under vacuum. Dried extracts will be stored at –80 °C until analysis. On the day of LC–MS, samples will be reconstituted in methanol and filtered (using a 0.22 µm PTFE filter) before injection. Chromatographic and mass spectrometric analyses will be performed using an Agilent UHPLC system coupled to a Bruker timsTOF Pro mass spectrometer equipped with an electrospray ionization (ESI) source.

***Plasma Hormones:*** For plasma steroid hormone analysis, 100 µL of plasma will be spiked with 10 µL of isotopically labelled internal standards (*d₃-testosterone, d₄-estradiol, d₉-progesterone,* and *d₄-cortisol*). Proteins will be precipitated with 200 µL cold methanol, vortexed, and centrifuged at 14,000 × g for 10 min at 4 °C. The supernatant will be loaded onto an Oasis PRiME HLB µElution plate, washed, and eluted with methanol:water (1:1) [13]. Hydroxysteroids will be derivatized with dansyl chloride, and ketosteroids with Girard T reagent, followed by incubation at 40 °C for 30 min and quenching with acetic acid. Derivatized samples will be analyzed by LC–MS (Agilent–Bruker timsTOF Pro) using a C18 column and a methanol–water gradient. Data will be processed in MetaboScape, and quantification performed using peak area ratios to deuterated internal standards.

***Urine Metabolomics:*** For urine metabolite analysis, the urine will be mixed with twice the volume of acetonitrile, vortexed for 30 seconds, and centrifuged at 14,000 × g for 10 minutes to precipitate proteins. The supernatant will be dried under vacuum and reconstituted in 2% acetonitrile [14]. For QC, 5 µL aliquots from each sample will be combined into a single pool. Each 100 µL sample or QC pool will be combined with of acetonitrile or water containing 5 µM CUDA (*12-(cyclohexylcarbamoyl)amino-dodecanoic acid*) as a non-endogenous internal standard for LC–MS normalization. After mixing at 1,000 rpm for 2 min at 4 °C, the samples will be centrifuged at 4,600 × g for 10 min. Then, 100 µL of the clarified supernatant will be transferred for LC–MS analysis. The CUDA spike-in ensures consistent ionization, retention time, and inter-sample comparability, in accordance with established guidelines for untargeted metabolomics.

***Metagenomics:*** To characterise the taxonomic community composition and identify the functional genetic potential of healthy adult gut microbiomes, shotgun metagenomics will be performed from stool samples collected from the five age cohorts. Briefly, total genomic DNA will be extracted from stool samples using an in-house developed protocol that maximises DNA yield and preserves taxonomic diversity and abundance as verified using a mock microbial community standard. Bacterial and fungal sequences will be obtained through the long-read Oxford Nanopore Technology (ONT) platform. Metagenomic Assembled Genomes (MAGs) will be reconstructed to gain insights into microbial community composition, relative abundance, and the presence or absence of metabolic pathways. Important metabolic pathways of microbial origin, such as those involved in fibre digestion, the synthesis of essential nutrients, and metabolic complementarity between members of the gut microbiota, will be elucidated. A primary objective of this analysis is to establish, for the first time, a baseline gut microbiome community for healthy adult Indians across all age cohorts, from young to old. Microbial abundance, community composition, and pathway completion will be investigated to draw correlations and perform enrichment analysis using metadata from volunteers, e.g., their diets, exercise regimes, socio-economic status, lifestyle, substance abuse, mental well-being, etc. Through a detailed questionnaire, data on the frequency of bowel movements and the consumption of probiotics and prebiotics will also be recorded. Furthermore, the gut microbiome abundance and metabolic repertoire will be assessed in relation to serum biomarkers reflecting inflammation, essential nutrients such as vitamins, and microbial metabolites, including SCFAs, which can have systemic consequences on an individual’s health. The data on dietary habits and lifestyle, collected from the study, will be used to perform correlation analysis of taxonomic and functional abundances of the gut microbiome with diet, lifestyle, and overall well-being.

***Hair Roughness:*** All hair specimens are biobanked at ambient temperature and then processed for surface roughness analysis using optical imaging techniques. The hair strands are mounted on a glass slide using tape at both ends. Mounted slides are imaged using an Olympus IX83 epifluorescence microscope under constant brightfield acquisition settings to ensure comparability across samples.

## ***Data Collection Methodology (Figure 3)***

### Literature Search Strategy

**PubMed search:** (“cohort study” OR “biobank” OR “population study”) AND (“multi-omics” OR “proteomics” OR “metabolomics” OR “genomics” OR “epigenomics” OR “metagenomics”) AND (“healthy” OR “population-based” OR “community”) AND (“aging” OR “adult” OR “lifespan”)

**Timeframe:** January 2015 - January 2025 (10 years)

**Additional sources:** Deep Research functionality from large language models such as Gemini/Claude were used to add additional cohorts from web searches that may have been missed from the literature review. The accuracy of information retrieved for each such identified cohort was independently validated by the authors.

### Inclusion Criteria

1. Recruited healthy volunteers OR population-based samples (not disease-specific cases only)
2. Generated at least one omics dataset from biological specimens
3. Published primary cohort description in peer-reviewed journal OR documented on official website/ bioRxiv.
4. Sample size N ≥ 100 participants
5. Active/ongoing or completed enrollment (not proposal-stage only)

## ***Supplementary Table 1: Complete List of Global Multi-Omics Cohorts (Figure 3)***

Complete dataset of 102 cohorts studying healthy populations worldwide, including geographic distribution, sample size and continent. An interactive version of Figure 3 can be found at <https://adb3502.github.io/Global-Multi-Omics-Cohort-Studies/>

| # | Cohort Name | Location | Country | Study Size | Continent |
| --- | --- | --- | --- | --- | --- |
| 1 | All of Us Research Program | National (340+ sites) | USA | 330,000 | North America |
| 2 | Human Phenome Project | Rehovot | Israel | 28,000 | Middle East |
| 3 | UK Biobank | National recruitment | United Kingdom | 500,000 | Europe |
| 4 | China Kadoorie Biobank | 10 regions (5 urban/5 rural) | China | 512,000 | Asia |
| 5 | Tohoku Medical Megabank | Miyagi and Iwate Prefectures | Japan | 157,602 | Asia |
| 6 | Genome India Project | National (20+ institutions) | India | 10,000 | Asia |
| 7 | FinnGen | Nationwide | Finland | 500,348 | Europe |
| 8 | Singapore NPM (PRECISE) | Singapore | Singapore | 10,000 | Asia |
| 9 | Taiwan Biobank | Nationwide | Taiwan | 151,406 | Asia |
| 10 | CanPath | 7 provinces (BC to Atlantic) | Canada | 330,000 | North America |
| 11 | Lifelines | Northern Netherlands | Netherlands | 167,729 | Europe |
| 12 | Estonian Biobank | Nationwide | Estonia | 210,000 | Europe |
| 13 | HUNT Study | Trøndelag County | Norway | 240,000 | Europe |
| 14 | NAKO German National Cohort | 18 centers (8 regional clusters) | Germany | 205,415 | Europe |
| 15 | deCODE genetics | Nationwide | Iceland | 160,000 | Europe |
| 16 | Korean Genome Epidemiology Study (KoGES) | Multiple sites | South Korea | 72,298 | Asia |
| 17 | Qatar Biobank | Nationwide | Qatar | 60,000 | Middle East |
| 18 | Australian 45 and Up Study | New South Wales | Australia | 267,000 | Oceania |
| 19 | Israel Psifas Virtual Biobank | Nationwide | Israel | 70,000 | Middle East |
| 20 | Saudi Human Genome Program | 15+ sites | Saudi Arabia | 100,000 | Middle East |
| 21 | UAE Emirati Genome Programme | Nationwide | United Arab Emirates | 500,000 | Middle East |
| 22 | Mexican Biobank | All 32 states (898 localities) | Mexico | 6,057 | Latin America |
| 23 | ELSA-Brasil | 6 cities | Brazil | 15,105 | Latin America |
| 24 | CÓDIGO | 14 populations | Colombia | 1,441 | Latin America |
| 25 | H3Africa AWI-Gen | Multi-site | South Africa/Ghana/Burkina Faso/Kenya | 12,000 | Africa |
| 26 | 54Gene Biobank | Lagos + 6 geopolitical zones | Nigeria | 75,000 | Africa |
| 27 | KEMRI Biobank | Kilifi + multiple sites | Kenya | 1,000,000 | Africa |
| 28 | Together for CHANGE | Multiple countries | Multi-national Africa | 20,000 | Africa |
| 29 | Generation Scotland | Nationwide | Scotland | 24,000 | Europe |
| 30 | Norwegian Mother Father Child Cohort (MoBa) | Nationwide | Norway | 114,500 | Europe |
| 31 | EPIC (European Prospective Investigation into Cancer) | 23 centers in 10 countries | Multi-national Europe | 521,000 | Europe |
| 32 | CoLaus | Lausanne | Switzerland | 6,738 | Europe |
| 33 | KORA | Augsburg region | Germany | 18,000 | Europe |
| 34 | Rotterdam Study | Rotterdam | Netherlands | 14,926 | Europe |
| 35 | TwinsUK | Nationwide | United Kingdom | 15,000 | Europe |
| 36 | Danish National Biobank | Nationwide | Denmark | 900,000 | Europe |
| 37 | Swedish CArdioPulmonary bioImage Study (SCAPIS) | 6 universities | Sweden | 30,154 | Europe |
| 38 | Airwave Health Monitoring Study | Nationwide | United Kingdom | 40,000 | Europe |
| 39 | Northern Sweden Health Disease Cohort | 4 cohorts | Sweden | 160,000 | Europe |
| 40 | French E3N Cohort | Nationwide | France | 98,995 | Europe |
| 41 | CONSTANCES | 22 health centers | France | 220,000 | Europe |
| 42 | Rhineland Study | Bonn region | Germany | 30,000 | Europe |
| 43 | UK ALSPAC | Bristol | United Kingdom | 14,500 | Europe |
| 44 | Genomics England 100000 Genomes | Nationwide | United Kingdom | 100,000 | Europe |
| 45 | Swiss Personalized Health Network | Multi-site | Switzerland | 35,000 | Europe |
| 46 | Belgium Biobank | Multi-site | Belgium | 50,000 | Europe |
| 47 | BBMRI-ERIC Network | 33 countries | Multi-national Europe | 100,000,000 | Europe |
| 48 | BioBank Japan | Nationwide | Japan | 270,000 | Asia |
| 49 | Nagahama Cohort | Nagahama City | Japan | 30,000 | Asia |
| 50 | China Health Retirement Longitudinal Study (CHARLS) | 28 provinces | China | 23,000 | Asia |
| 51 | Shanghai Changfeng Study | Shanghai | China | 10,000 | Asia |
| 52 | Kailuan Study | Tangshan | China | 101,510 | Asia |
| 53 | Taizhou Longitudinal Study | Taizhou | China | 120,000 | Asia |
| 54 | China Multi-Ethnic Cohort (CMEC) | Southwest China | China | 100,000 | Asia |
| 55 | Singapore Chinese Health Study | Singapore | Singapore | 63,000 | Asia |
| 56 | Singapore Malay Eye Study | Singapore | Singapore | 3,280 | Asia |
| 57 | Multi-Ethnic Cohort (MEC) | Hawaii/California | USA | 215,000 | North America |
| 58 | Framingham Heart Study | Framingham MA | USA | 15,000 | North America |
| 59 | Jackson Heart Study | Jackson MS | USA | 5,306 | North America |
| 60 | Women’s Health Initiative | 40 centers | USA | 161,808 | North America |
| 61 | Nurses’ Health Study | Nationwide | USA | 280,000 | North America |
| 62 | Black Women’s Health Study | Nationwide | USA | 59,000 | North America |
| 63 | Hispanic Community Health Study | 4 centers | USA | 16,415 | North America |
| 64 | Strong Heart Study | 13 tribes | USA | 4,549 | North America |
| 65 | PAGE Study | Multi-site | USA | 50,000 | North America |
| 66 | BioMe Biobank | New York | USA | 55,000 | North America |
| 67 | UCLA ATLAS Community Health | Los Angeles | USA | 150,000 | North America |
| 68 | Colorado Center for Personalized Medicine | Colorado | USA | 200,000 | North America |
| 69 | Geisinger MyCode | Pennsylvania | USA | 300,000 | North America |
| 70 | Mount Sinai BioMe | New York | USA | 55,000 | North America |
| 71 | eMERGE Network | Multiple sites | USA | 100,000 | North America |
| 72 | TOPMed | Multiple sites | USA | 180,000 | North America |
| 73 | CARTaGENE | Quebec | Canada | 43,000 | North America |
| 74 | Ontario Health Study | Ontario | Canada | 225,000 | North America |
| 75 | BC Generations Project | British Columbia | Canada | 30,000 | North America |
| 76 | Alberta’s Tomorrow Project | Alberta | Canada | 55,000 | North America |
| 77 | Atlantic PATH | Atlantic provinces | Canada | 35,000 | North America |
| 78 | Maelstrom Research | Pan-Canadian coordination | Canada | 2,000,000 | North America |
| 79 | Personalized Risk Stratification for CVD (PERSIC) | Quebec | Canada | 8,000 | North America |
| 80 | GenomeAsia 100K | Multiple Asian countries | Multi-national Asia | 100,000 | Asia |
| 81 | PI-Hub China | Multiple provinces | China | 200,000 | Asia |
| 82 | Biobank Thailand | Nationwide | Thailand | 50,000 | Asia |
| 83 | Vietnam Biobank Initiative | Hanoi/Ho Chi Minh | Vietnam | 30,000 | Asia |
| 84 | Malaysian Cohort | Multiple states | Malaysia | 106,000 | Asia |
| 85 | Indonesia Biobank | Jakarta + regions | Indonesia | 50,000 | Asia |
| 86 | iPOP (Integrative Personal Omics Profile) | Stanford | USA | 100 | North America |
| 87 | 1000 Immunomes Project (1KIP) | Stanford | USA | 1,001 | North America |
| 88 | Arivale | Seattle | USA | 5,000 | North America |
| 89 | ABIOS (Ageing BIOmarker Study) | Singapore | Singapore | 1,500 | Asia |
| 90 | **BHARAT Study (Longevity India)** | **Bangalore** | **India** | **5,000** | **Asia** |
| 91 | MARK-AGE Project | Multi-site | Europe | 3,337 | Europe |
| 92 | CSIR Phenome India (PI-CHeCK) | Multi-site | India | 10,000 | Asia |
| 93 | Singapore Longitudinal Ageing Study (SLAS) | Singapore | Singapore | 6,000 | Asia |
| 94 | AIBL (Australian Imaging Biomarkers Lifestyle) | Melbourne | Australia | 3,045 | Oceania |
| 95 | Kaiser Healthy Aging and Diverse Life Experiences (KHANDLE) | San Francisco Bay Area | USA | 1,800 | North America |
| 96 | Berlin Aging Study II (BASE-II) | Berlin | Germany | 2,200 | Europe |
| 97 | HEARTS Sweden | Gothenburg | Sweden | 5,913 | Europe |
| 98 | SNAC-K (Swedish National study on Aging and Care) | Stockholm | Sweden | 3,363 | Europe |
| 99 | Longitudinal Aging Study Amsterdam (LASA) | Amsterdam | Netherlands | 3,107 | Europe |
| 100 | Australian Longitudinal Study of Ageing (ALSA) | Adelaide | Australia | 2,087 | Oceania |
| 101 | Baltimore Longitudinal Study of Aging (BLSA) | Baltimore | USA | 3,000 | North America |
| 102 | Health and Retirement Study (HRS) | National | USA | 20,000 | North America |

## ***Detailed Scoring Criteria Definitions (Figure 4)***

### 1. Omics Coverage (0-5 scale)

Assessment of breadth and depth of molecular profiling platforms.

**0:** No omics data

**1:** Single omics, limited depth (e.g., genotyping arrays <500K SNPs)

**2:** Single omics, high depth (WGS/WES) OR 2 layers, limited depth

**3:** 2-3 omics layers with substantial depth (e.g., WGS + proteomics OR metabolomics + transcriptomics + epigenomics)

**4:** 4-5 distinct omics layers (e.g., genomics + proteomics + metabolomics + transcriptomics)

**5:** 6+ comprehensive omics layers (genomics, transcriptomics, proteomics, metabolomics, epigenomics, metagenomics) with discovery-driven approaches

### 2. Clinical/Social Metadata (0-5 scale)

Assessment of phenotypic data breadth and depth beyond molecular measurements.

**0:** No metadata

**1:** Demographics only (age, sex, ancestry)

**2:** Basic clinical (blood pressure, BMI, routine blood tests) + limited surveys

**3:** Comprehensive clinical OR comprehensive social, but not both

**4:** Comprehensive clinical + comprehensive social + family history

**5:** Exceptional depth - comprehensive clinical + social + environmental exposures + EHR linkage + imaging + longitudinal follow-up

### 3. Digital Health Tools (0-5 scale)

Assessment of digital data capture and real-time/continuous monitoring infrastructure.

**0:** None (paper-based)

**1:** Electronic data capture only (web forms, tablets)

**2:** EHR integration OR basic digital tools (single wearable type)

**3:** EHR + some wearables/apps OR continuous monitoring device

**4:** Comprehensive EHR + multiple wearables + smartphone apps

**5:** Advanced digital ecosystem (continuous glucose monitors, multiple wearables, real-time monitoring, AI-driven platforms, passive sensing)

### 4. Demographic Coverage (0-5 scale)

Assessment of population diversity and representativeness across ancestry, age, sex, geography, and socioeconomic strata.

**0:** Very limited (single ethnicity >95%, narrow age range <10 years, single sex)

**1:** Limited (>80% one ethnicity, narrow age range, or single sex)

**2:** Moderate (2-3 ethnicities OR good age range 30+ years but limited ethnic diversity)

**3:** Good (multiple ethnicities, broad age range 30+ years, both sexes balanced, some geographic diversity)

**4:** Excellent (many ethnicities/ancestries, multi-generational, both sexes, urban/rural representation, multiple regions)

**5:** Exceptional (explicitly designed for diversity, >5 ethnicities/ancestries, full age spectrum including children/elderly, underrepresented populations prioritized, rural/urban/socioeconomic strata captured)

### 5. Cohort Size (0-5 scale)

Assessment of sample size enrolled or targeted for enrollment.

**0:** <100 participants

**1:** 100-1,000

**2:** 1,001-10,000

**3:** 10,001-50,000

**4:** 50,001-200,000

**5:** >200,000

## ***Supplementary Table 2: Multi-Omics Cohort Scoring - Detailed Criteria and Justifications (Figure 4)***

Scoring of region-wise representative worldwide multi-omics cohorts across five dimensions: omics coverage, clinical/social metadata, digital health tools, demographic coverage, and cohort size. Each category scored 0-5 based on standardized criteria mentioned above.

| Cohort | Country | N | Omics Coverage (0-5) | Clinical/Social Metadata (0-5) | Digital Health Tools (0-5) | Demographic Coverage (0-5) | Cohort Size (0-5) | Total Score | Notes |
| --- | --- | --- | --- | --- | --- | --- | --- | --- | --- |
| **UK Biobank** | UK | 500,000 | 5 | 5 | 3 | 2 | 5 | 20/25 | Genomics (WGS subset), proteomics (Olink), metabolomics (NMR), imaging (MRI); EHR linkage, wearables; predominantly European but includes minorities |
| **All of Us** | USA | 245,388 | 3 | 5 | 4 | 5 | 5 | 22/25 | Genomics (WGS), proteomics (planned), metabolomics (planned); designed for diversity (77% underrepresented); comprehensive EHR + Fitbit |
| **ABIOS** | Singapore | ~5000 | 4 | 4 | 2 | 4 | 2 | 16/25 | Epigenomics (850K array), proteomics (SomaScan 7K), metabolomics (LC-MS), lipidomics; Asian population focus; comprehensive clinical data capture |
| **Lifelines** | Netherlands | 167,000 | 4 | 5 | 3 | 2 | 4 | 18/25 | Genomics, proteomics (limited), metabolomics; three-generation design; comprehensive questionnaires; predominantly Dutch |
| **BHARAT Study** | India | 5,000 | 5 | 4 | 2 | 5 | 2 | 18/25 | **Epigenomics** (MSA 270K), **SNP genotyping** (APMRA 750K), **proteomics** (LC-MS untargeted), **metabolomics** (LC-MS plasma/urine), **lipidomics** (LC-MS), **metagenomics** (ONT shotgun), **immunophenotyping** (flow cytometry); comprehensive clinical + social + dietary metadata; electronic data capture; designed for Indian diversity (rural-urban, vegetarian/non-vegetarian, 5 age strata, balanced sex); moderate size |
| **Human Phenome Project** | Israel | 28000 | 5 | 5 | 5 | 3 | 4 | 22/25 | 7+ omics layers, comprehensive blood tests, anthropometrics, imaging, cognitive assessments, longitudinal follow-up. CGM monitoring, sleep tracking, wearables, mobile app integration. Israeli Jewish and Arab populations; single country but multiple ethnic groups |
| **KEMRI Biobank** | Kenya | 300000 | 3 | 5 | 4 | 3 | 5 | 20/25 | Genotyping >5,000 Biobank >1M samples; expanding multi-omics capability, Real-time morbidity surveillance system; digital data collection infrastructure |
| **H3Africa AWI-Gen** | Sub-Saharan Africa | 10500 | 4 | 5 | 2 | 4 | 3 | 18/25 | Genotyping ~10,700 (H3Africa array); WGS ~160; microbiome in subsets. Blood biomarkers, anthropometrics, lifestyle, point-of-care tests, social determinants,  Clinic-based; limited digital tools; expanding capabilities |
| **ELSA-Brazil** | Brazil | 15105 | 3 | 5 | 3 | 3 | 3 | 17/25 | Comprehensive CV phenotyping, cognitive assessments, imaging, 5-year follow-up, social determinants, Accelerometry in subsets; digital health expansion underway |
| **BLSA** | USA | 1200 | 4 | 5 | 2 | 2 | 2 | 15/25 | Proteomics (SASP panel N=1,060), metabolomics (N=252), genetics; deep phenotyping focus, 3-day visits, imaging, cognition, physical function, decades follow-up, Baltimore region; historically White; recent diversification efforts |

References

1.	Goldberg DC, Cloud C, Lee SM, Barnes B, Gruber S, Kim E, Pottekat A, Westphal MS, McAuliffe L, Majounie E, Kalayil Manian M, Zhu Q, Tran C, et al. Scalable screening of ternary-code DNA methylation dynamics associated with human traits. Cell Genom. 2025; 5:100929.

<https://doi.org/10.1016/j.xgen.2025.100929> PMID:[40614726](https://pubmed.ncbi.nlm.nih.gov/40614726)

2.	Zhou W, Triche TJ Jr, Laird PW, Shen H. SeSAMe: reducing artifactual detection of DNA methylation by Infinium BeadChips in genomic deletions. Nucleic Acids Res. 2018; 46:e123.

<https://doi.org/10.1093/nar/gky691> PMID:[30085201](https://pubmed.ncbi.nlm.nih.gov/30085201)

3.	Horvath S. DNA methylation age of human tissues and cell types. Genome Biol. 2013; 14:R115.

<https://doi.org/10.1186/gb-2013-14-10-r115> PMID:[24138928](https://pubmed.ncbi.nlm.nih.gov/24138928)

4.	Hannum G, Guinney J, Zhao L, Zhang L, Hughes G, Sadda S, Klotzle B, Bibikova M, Fan JB, Gao Y, Deconde R, Chen M, Rajapakse I, et al. Genome-wide methylation profiles reveal quantitative views of human aging rates. Mol Cell. 2013; 49:359–67.

<https://doi.org/10.1016/j.molcel.2012.10.016> PMID:[23177740](https://pubmed.ncbi.nlm.nih.gov/23177740)

5.	Lu AT, Quach A, Wilson JG, Reiner AP, Aviv A, Raj K, Hou L, Baccarelli AA, Li Y, Stewart JD, Whitsel EA, Assimes TL, Ferrucci L, Horvath S. DNA methylation GrimAge strongly predicts lifespan and healthspan. Aging (Albany NY). 2019; 11:303–27.

<https://doi.org/10.18632/aging.101684> PMID:[30669119](https://pubmed.ncbi.nlm.nih.gov/30669119)

6.	Levine ME, Lu AT, Quach A, Chen BH, Assimes TL, Bandinelli S, Hou L, Baccarelli AA, Stewart JD, Li Y, Whitsel EA, Wilson JG, Reiner AP, et al. An epigenetic biomarker of aging for lifespan and healthspan. Aging (Albany NY). 2018; 10:573–91.

<https://doi.org/10.18632/aging.101414> PMID:[29676998](https://pubmed.ncbi.nlm.nih.gov/29676998)

7.	Belsky DW, Caspi A, Corcoran DL, Sugden K, Poulton R, Arseneault L, Baccarelli A, Chamarti K, Gao X, Hannon E, Harrington HL, Houts R, Kothari M, et al. DunedinPACE, a DNA methylation biomarker of the pace of aging. Elife. 2022; 11:e73420.

<https://doi.org/10.7554/eLife.73420> PMID:[35029144](https://pubmed.ncbi.nlm.nih.gov/35029144)

8.	Verlouw JA, Clemens E, de Vries JH, Zolk O, Verkerk AJ, Am Zehnhoff-Dinnesen A, Medina-Gomez C, Lanvers-Kaminsky C, Rivadeneira F, Langer T, van Meurs JB, van den Heuvel-Eibrink MM, Uitterlinden AG, Broer L. A comparison of genotyping arrays. Eur J Hum Genet. 2021; 29:1611–24.

<https://doi.org/10.1038/s41431-021-00917-7> PMID:[34140649](https://pubmed.ncbi.nlm.nih.gov/34140649)

9.	Balagué-Dobón L, Cáceres A, González JR. Fully exploiting SNP arrays: a systematic review on the tools to extract underlying genomic structure. Brief Bioinform. 2022; 23:bbac043.

<https://doi.org/10.1093/bib/bbac043> PMID:[35211719](https://pubmed.ncbi.nlm.nih.gov/35211719)

10.	Lu YH, Wang BH, Jiang F, Mo XB, Wu LF, He P, Lu X, Deng FY, Lei SF. Multi-omics integrative analysis identified SNP-methylation-mRNA: Interaction in peripheral blood mononuclear cells. J Cell Mol Med. 2019; 23:4601–10.

<https://doi.org/10.1111/jcmm.14315>
PMID:[31106970](https://pubmed.ncbi.nlm.nih.gov/31106970)

11.	Ulmer CZ, Jones CM, Yost RA, Garrett TJ, Bowden JA. Optimization of Folch, Bligh-Dyer, and Matyash sample-to-extraction solvent ratios for human plasma-based lipidomics studies. Anal Chim Acta. 2018; 1037:351–7.

<https://doi.org/10.1016/j.aca.2018.08.004> PMID:[30292311](https://pubmed.ncbi.nlm.nih.gov/30292311)

12.	Matyash V, Liebisch G, Kurzchalia TV, Shevchenko A, Schwudke D. Lipid extraction by methyl-tert-butyl ether for high-throughput lipidomics. J Lipid Res. 2008; 49:1137–46.

<https://doi.org/10.1194/jlr.D700041-JLR200> PMID:[18281723](https://pubmed.ncbi.nlm.nih.gov/18281723)

13.	Juck M, Long W. Determination of Hormones in Serum by LC / MS / MS Using Agilent Bond Elut Plexa SPE Application Note. Agilent Technologies. 2017; 2:1–12.

14.	Southam AD, Haglington LD, Najdekr L, Jankevics A, Weber RJ, Dunn WB. Assessment of human plasma and urine sample preparation for reproducible and high-throughput UHPLC-MS clinical metabolic phenotyping. Analyst. 2020; 145:6511–23.

<https://doi.org/10.1039/d0an01319f>
PMID:[32760982](https://pubmed.ncbi.nlm.nih.gov/32760982)
