# Supplementary material for: The BHARAT study: a multi-modal, multi-omics investigation of aging signatures in the Indian population
Source: Aging (Albany NY). 2026 Apr 24;18(1):380–96. doi: 10.18632/aging.206373 (PMC13632655; doi:10.18632/aging.206373)
Supplement: Supplementary file 2 [file aging-18-1-206373-s002.docx]

**Main Authors**

| **Author Name** | **Affiliations** | **Email** |
| --- | --- | --- |
| Suramya Asthana | ^1^Longevity India, Division of Interdisciplinary Sciences, IISc, Bengaluru 560012  ^2^Department of Developmental Biology and Genetics, Division of Biological Sciences, IISc, Bengaluru 560012 | suramyaa@iisc.ac.in |
| Amruth Deepak Bhat | ^1^Longevity India, Division of Interdisciplinary Sciences, IISc, Bengaluru 560012  ^3^Department of Bioengineering, Division of Interdisciplinary Sciences, IISc, Bengaluru 560012 | amruthbhat@iisc.ac.in |
| Gayathri Mahadevan | ^1^Longevity India, Division of Interdisciplinary Sciences, IISc, Bengaluru 560012  ^2^Department of Developmental Biology and Genetics, Division of Biological Sciences, IISc, Bengaluru 560012 | mgayathri@iisc.ac.in |
| Lakshmi Kothegala | ^1^Longevity India, Division of Interdisciplinary Sciences, IISc, Bengaluru 560012  ^2^Department of Developmental Biology and Genetics, Division of Biological Sciences, IISc, Bengaluru 560012 | lakshmikv@iisc.ac.in |
| Swati Negi | ^1^Longevity India, Division of Interdisciplinary Sciences, IISc, Bengaluru 560012  ^2^Department of Developmental Biology and Genetics, Division of Biological Sciences, IISc, Bengaluru 560012 | swatinegi@iisc.ac.in |
| Seema Yadav | ^1^Longevity India, Division of Interdisciplinary Sciences, IISc, Bengaluru 560012  ^2^Department of Developmental Biology and Genetics, Division of Biological Sciences, IISc, Bengaluru 560012 | seema2024@iisc.ac.in |
| Anupama Sudhakaran | ^1^Longevity India, Division of Interdisciplinary Sciences, IISc, Bengaluru 560012  ^2^Department of Developmental Biology and Genetics, Division of Biological Sciences, IISc, Bengaluru 560012 | anupamas2@iisc.ac.in |
| Annwesha Roy | ^1^Longevity India, Division of Interdisciplinary Sciences, IISc, Bengaluru 560012  ^2^Department of Developmental Biology and Genetics, Division of Biological Sciences, IISc, Bengaluru 560012 | annwesharoy@iisc.ac.in |
| Meet Makwana | ^1^Longevity India, Division of Interdisciplinary Sciences, IISc, Bengaluru 560012  ^2^Department of Developmental Biology and Genetics, Division of Biological Sciences, IISc, Bengaluru 560012 | meetchetanb1@iisc.ac.in |
| Rahul Patel | ^1^Longevity India, Division of Interdisciplinary Sciences, IISc, Bengaluru 560012  ^2^Department of Developmental Biology and Genetics, Division of Biological Sciences, IISc, Bengaluru 560012 | rahulprakas1@iisc.ac.in |
| Harshavardhan Rao B | ^4^Department of Gastroenterology, MS Ramaiah Medical College, RUAS, Bengaluru 560054 | harshavardhanrao1985  @gmail.com |
| Caroline Elizabeth George | ^5^Community Health, Palliative Care & Research Division, Bangalore Baptist Hospital, Bengaluru 560032 | carolinelizabethj  @gmail.com |
| Srimonta Gayen | ^1^Longevity India, Division of Interdisciplinary Sciences, IISc, Bengaluru 560012  ^2^Department of Developmental Biology and Genetics, Division of Biological Sciences, IISc, Bengaluru 560012 | srimonta@iisc.ac.in |
| Shantanu Shukla | ^1^Longevity India, Division of Interdisciplinary Sciences, IISc, Bengaluru 560012  ^2^Department of Developmental Biology and Genetics, Division of Biological Sciences, IISc, Bengaluru 560012 | sps@iisc.ac.in |
| Siddharth Jhunjhunwala | ^1^Longevity India, Division of Interdisciplinary Sciences, IISc, Bengaluru 560012  ^3^Department of Bioengineering, Division of Interdisciplinary Sciences, IISc, Bengaluru 560012 | siddharth@iisc.ac.in |
| Prosenjit Sen | ^1^Longevity India, Division of Interdisciplinary Sciences, IISc, Bengaluru 560012  ^6^Centre for Nano Science and Engineering, Division of Interdisciplinary Sciences, IISc, Bengaluru 560012 | prosenjits@iisc.ac.in |
| Prabhdeep Kaur | ^1^Longevity India, Division of Interdisciplinary Sciences, IISc, Bengaluru 560012  ^7^Isaac Centre for Public Health, Tata IISc Medical School, IISc, Bengaluru 560012 | pkaur@iisc.ac.in |
| Ramray Bhat | ^1^Longevity India, Division of Interdisciplinary Sciences, IISc, Bengaluru 560012  ^2^Department of Developmental Biology and Genetics, Division of Biological Sciences, IISc, Bengaluru 560012  ^3^Department of Bioengineering, Division of Interdisciplinary Sciences, IISc, Bengaluru 560012 | ramray@iisc.ac.in |
| Deepak Kumar Saini | ^1^Longevity India, Division of Interdisciplinary Sciences, IISc, Bengaluru 560012  ^2^Department of Developmental Biology and Genetics, Division of Biological Sciences, IISc, Bengaluru 560012  ^3^Department of Bioengineering, Division of Interdisciplinary Sciences, IISc, Bengaluru 560012 | deepaksaini@iisc.ac.in |

**Contributing Authors**

| **Name** | **Affiliation** | **Email** |
| --- | --- | --- |
| ***Longevity India Team*** | | |
| Ambarish Ghosh | ^1^Longevity India, Division of Interdisciplinary Sciences, Indian Institute of Science, Bengaluru 560012  ^6^Centre for Nano Science and Engineering, Division of Interdisciplinary Sciences, Indian Institute of Science, Bengaluru 560012  ^8^Department of Physics, Indian Institute of Science, Bangalore 560012, India | ambarish@iisc.ac.in |
| Ananya Sarkar | ^1^Longevity India, Division of Interdisciplinary Sciences, Indian Institute of Science, Bengaluru 560012  ^2^Department of Developmental Biology and Genetics, Division of Biological Sciences, Indian Institute of Science, Bengaluru 560012 | sananya@iisc.ac.in |
| Anchita Gopikrishnan | ^1^Longevity India, Division of Interdisciplinary Sciences, Indian Institute of Science, Bengaluru 560012  ^2^Department of Developmental Biology and Genetics, Division of Biological Sciences, Indian Institute of Science, Bengaluru 560012 | anchitag@iisc.ac.in |
| Annapoorni Rangarajan | ^1^Longevity India, Division of Interdisciplinary Sciences, Indian Institute of Science, Bengaluru 560012  ^2^Department of Developmental Biology and Genetics, Division of Biological Sciences, Indian Institute of Science, Bengaluru 560012 | anu@iisc.ac.in |
| Anuva R | ^1^Longevity India, Division of Interdisciplinary Sciences, Indian Institute of Science, Bengaluru 560012  ^9^Department of Microbiology and Cell Biology, Division of Biological Sciences, Indian Institute of Science, Bengaluru 560012 | anuvar@iisc.ac.in |
| Arvind Ramanathan | ^15^Institute for Stem Cell Science and Regenerative Medicine (InStem), Bangalore Life Science Cluster, Bengaluru 560065 | arvind@instem.res.in |
| Ayushi Verma | ^1^Longevity India, Division of Interdisciplinary Sciences, Indian Institute of Science, Bengaluru 560012  ^2^Department of Developmental Biology and Genetics, Division of Biological Sciences, Indian Institute of Science, Bengaluru 560012 | ayushiv@iisc.ac.in |
| Bishal Singh | ^1^Longevity India, Division of Interdisciplinary Sciences, Indian Institute of Science, Bengaluru 560012  ^9^Department of Microbiology and Cell Biology, Division of Biological Sciences, Indian Institute of Science, Bengaluru 560012 | bishalsingh@iisc.ac.in |
| Chiranjib Bhattacharya | ^1^Longevity India, Division of Interdisciplinary Sciences, Indian Institute of Science, Bengaluru 560012  ^14^Department of Computer Science and Automation, Indian Institute of Science, Bengaluru 560012 | chiru@iisc.ac.in |
| Inbasekar C | ^1^Longevity India, Division of Interdisciplinary Sciences, Indian Institute of Science, Bengaluru 560012  ^2^Department of Developmental Biology and Genetics, Division of Biological Sciences, Indian Institute of Science, Bengaluru 560012 | inbasekarc@iisc.ac.in |
| Jalak Mehta | ^1^Longevity India, Division of Interdisciplinary Sciences, Indian Institute of Science, Bengaluru 560012  ^2^Department of Developmental Biology and Genetics, Division of Biological Sciences, Indian Institute of Science, Bengaluru 560012 | jalakmehta@iisc.ac.in |
| Jayant Sehgal | ^1^Longevity India, Division of Interdisciplinary Sciences, Indian Institute of Science, Bengaluru 560012  ^2^Department of Developmental Biology and Genetics, Division of Biological Sciences, Indian Institute of Science, Bengaluru 560012 | jayantsehgal@iisc.ac.in |
| Kaushik Chatterjee | ^1^Longevity India, Division of Interdisciplinary Sciences, Indian Institute of Science, Bengaluru 560012  ^3^Department of Bioengineering, Division of Interdisciplinary Sciences, Indian Institute of Science, Bengaluru 560012  ^10^Department of Materials Engineering, Indian Institute of Science, Bengaluru 560012 | kchatterjee@iisc.ac.in |
| Mohit Kumar Jolly | ^1^Longevity India, Division of Interdisciplinary Sciences, Indian Institute of Science, Bengaluru 560012  ^3^Department of Bioengineering, Division of Interdisciplinary Sciences, Indian Institute of Science, Bengaluru 560012 | mkjolly@iisc.ac.in |
| Narendra Dixit | ^1^Longevity India, Division of Interdisciplinary Sciences, Indian Institute of Science, Bengaluru 560012  ^3^Department of Bioengineering, Division of Interdisciplinary Sciences, Indian Institute of Science, Bengaluru 560012  ^11^Department of Chemical Engineering, Indian Institute of Science, Bengaluru 560012 | narendra@iisc.ac.in |
| Nikhil Gandasi | ^1^Longevity India, Division of Interdisciplinary Sciences, Indian Institute of Science, Bengaluru 560012  ^2^Department of Developmental Biology and Genetics, Division of Biological Sciences, Indian Institute of Science, Bengaluru 560012 | grnikhil@iisc.ac.in |
| Nischitha Baliga | ^1^Longevity India, Division of Interdisciplinary Sciences, Indian Institute of Science, Bengaluru 560012  ^9^Department of Microbiology and Cell Biology, Division of Biological Sciences, Indian Institute of Science, Bengaluru 560012 | nischithab@iisc.ac.in |
| Sagarika Prabhakar | ^1^Longevity India, Division of Interdisciplinary Sciences, Indian Institute of Science, Bengaluru 560012  ^2^Department of Developmental Biology and Genetics, Division of Biological Sciences, Indian Institute of Science, Bengaluru 560012 | sagarikap@iisc.ac.in |
| Sanjeeva Srivastava | ^16^Department of Biosciences and Bioengineering, Indian Institute of Technology Bombay, Mumbai 400076 | sanjeeva@iitb.ac.in |
| Sanjiv Sambandan | ^1^Longevity India, Division of Interdisciplinary Sciences, Indian Institute of Science, Bengaluru 560012  ^13^Instrumentation and Applied Physics, Indian Institute of Science, Bengaluru 560012 | sanjiv@iisc.ac.in |
| Shovamayee Maharana | ^1^Longevity India, Division of Interdisciplinary Sciences, Indian Institute of Science, Bengaluru 560012  ^9^Department of Microbiology and Cell Biology, Division of Biological Sciences, Indian Institute of Science, Bengaluru 560012 | shova@iisc.ac.in |
| Shweta Thakar | ^1^Longevity India, Division of Interdisciplinary Sciences, Indian Institute of Science, Bengaluru 560012  ^2^Department of Developmental Biology and Genetics, Division of Biological Sciences, Indian Institute of Science, Bengaluru 560012 | shwetapraka1@iisc.ac.in |
| Subba Rao GS | ^1^Longevity India, Division of Interdisciplinary Sciences, Indian Institute of Science, Bengaluru 560012  ^9^Department of Microbiology and Cell Biology, Division of Biological Sciences, Indian Institute of Science, Bengaluru 560012 | subba@iisc.ac.in |
| Swaminathan S | ^1^Longevity India, Division of Interdisciplinary Sciences, Indian Institute of Science, Bengaluru 560012  ^3^Department of Bioengineering, Division of Interdisciplinary Sciences, Indian Institute of Science, Bengaluru 560012  ^12^Department of Nephrology, Tata IISc Medical School, Indian Institute of Science, Bengaluru | swami@iisc.ac.in |
| Tavpritesh Sethi | ^17^Computational Biology, Indraprastha Institute of Information Technology, Delhi 110020 | tavpriteshsethi@iiitd.ac.in |
| Vinod Bhat | ^1^Longevity India, Division of Interdisciplinary Sciences, Indian Institute of Science, Bengaluru 560012  ^9^Department of Microbiology and Cell Biology, Division of Biological Sciences, Indian Institute of Science, Bengaluru 560012 | vinod.bhat@fsid-iisc.in |
| Vishal Rao US | ^18^Department of Head and Neck Oncology, HCG Cancer Center, Bengaluru 560027 | drvishalrao@gmail.com |
| Yogesh | ^1^Longevity India, Division of Interdisciplinary Sciences, Indian Institute of Science, Bengaluru 560012  ^2^Department of Developmental Biology and Genetics, Division of Biological Sciences, Indian Institute of Science, Bengaluru 560012 | yogesh1@iisc.ac.in |
| ***Clinical Partners*** | | |
| Maddipati Tatarao | ^5^Community Health, Palliative Care & Research Division, Bangalore Baptist Hospital, Bengaluru 560032 | tatarao777@gmail.com |
| Suman Sarah Varghese | ^5^Community Health, Palliative Care & Research Division, Bangalore Baptist Hospital, Bengaluru 560032 | sumansarah.92@gmail.com |
| Manjula KS | ^5^Community Health, Palliative Care & Research Division, Bangalore Baptist Hospital, Bengaluru 560032 | manjulaks2014@gmail.com |
| S Praveena | ^5^Community Health, Palliative Care & Research Division, Bangalore Baptist Hospital, Bengaluru 560032 | praveebio@gmail.com |
| Sharon Rimai | ^5^Community Health, Palliative Care & Research Division, Bangalore Baptist Hospital, Bengaluru 560032 | sharonrimai1996@gmail.com |
| Shreya Thadigotla | ^4^Department of Gastroenterology, MS Ramaiah Medical College, RUAS, Bengaluru 560054 | tshreyareddy20@gmail.com |
| Prateek Reddy | ^4^Department of Gastroenterology, MS Ramaiah Medical College, RUAS, Bengaluru 560054 | prateekreddyt@gmail.com |
| Drashti Godhani | ^4^Department of Gastroenterology, MS Ramaiah Medical College, RUAS, Bengaluru 560054 | drastigodhani@gmail.com |
| Anagha Pritvi Raj | ^4^Department of Gastroenterology, MS Ramaiah Medical College, RUAS, Bengaluru 560054 | anagharaj45@gmail.com |
| Bhoomika Y. | ^4^Department of Gastroenterology, MS Ramaiah Medical College, RUAS, Bengaluru 560054 | bhoomika2708@gmail.com |
| Harshit V. | ^4^Department of Gastroenterology, MS Ramaiah Medical College, RUAS, Bengaluru 560054 | harshith27v@gmail.com |
| Sathish Babu K.N. | ^19^Sri Madhusudan Sai Institute of Medical Sciences and Research, Chikkaballapur 562103 | satish.babu@smsimsr.org |
| Phaneendra Mallekavu | ^19^Sri Madhusudan Sai Institute of Medical Sciences and Research, Chikkaballapur 562103 | phaneendra.ms@smsimsr.org |
| Anil S. Bilimale | ^20^School of Public Health, JSS Medical College, JSS AHER, Mysuru 570015 | anilbilimale@jssuni.edu.in |
| Sheeba B | ^20^School of Public Health, JSS Medical College, JSS AHER, Mysuru 570015 | sheebab@jssuni.edu.in |
| Divya K L | ^20^School of Public Health, JSS Medical College, JSS AHER, Mysuru 570015 | divyakl@jssuni.edu.in |
| Hemanth R | ^20^School of Public Health, JSS Medical College, JSS AHER, Mysuru 570015 | hemanthhemi902@gmail.com |
| Aishwarya N | ^20^School of Public Health, JSS Medical College, JSS AHER, Mysuru 570015 | aishwaryanagesh@icloud.com |
| Shreya Gudi | ^21^Department of Geriatric Medicine, Bangalore Medical College & Research Institute, Bengaluru 560002 | gudishreya@gmail.com |
| Raghvendra BM | ^21^Department of Geriatric Medicine, Bangalore Medical College & Research Institute, Bengaluru 560002 | drrgbm78@gmail.com |
| Amit Bahuguna | ^22^Command Hospital, Bengaluru 560007 | afmcrocks@gmail.com |
| D Navya | ^22^Command Hospital, Bengaluru 560007 | navya.donaparthi@gmail.com |
| Devyani Sapra | ^22^Command Hospital, Bengaluru 560007 | dvyniz@gmail.com |
| Ashwin P Nair | ^22^Command Hospital, Bengaluru 560007 | ashwinktm@yahoo.com |
| Vishal Gautam | ^22^Command Hospital, Bengaluru 560007 | vishalgautam115@gmail.com |
| ***Industry Partners*** | | |
| Jagan L | ^23^Centenarians Life Sciences Pvt. Ltd. (Decode Age) | jagan.l@decodeage.com |
| Aditi Muglikar | ^23^Centenarians Life Sciences Pvt. Ltd. (Decode Age) | aditi.m@decodeage.com |
| Darshit Patel | ^23^Centenarians Life Sciences Pvt. Ltd. (Decode Age) | darshit@decodeage.com |
| John Mulder | ^24^Diagnoptics Technologies b.v. | j.mulder@diagnoptics.com |
| Tushar Asthana | ^25^ARCA AI | tushar@arcaai.com |
| Shibu V Ignatious | ^25^ARCA AI | dr.shibu@arcaai.com |
| Ramya Satish Kumar | ^25^ARCA AI | ramya@arcaai.com |
| Karthik Iyer | ^25^ARCA AI | karthik@arcaai.com |
| Rohit Rajan | ^25^ARCA AI | rohit@arcaai.com |
| Gaurav Agarwal | ^26^TATA 1mg Labs | gaurava@1mg.com |
| Shanmugam RK | ^26^TATA 1mg Labs | shanmugam.rk@1mg.com |
| Prashant Nag | ^26^TATA 1mg Labs | prashant.nag@1mg.com |
| Arpita Roy Dam | ^26^TATA 1mg Labs | Arpita.dam@1mg.com |
| Vinisha Nahata | ^26^TATA 1mg Labs | vinisha.nahata@1mg.com |
| Swati Gupta | ^26^TATA 1mg Labs | Swati.gupta@1mg.com |
| Vandana Lal | ^27^Lal Path Labs | vandana.lal@lalpathlabs.com |
| Alok Sharma | ^27^Lal Path Labs | alok.sharma@lalpathlabs.com |
| Archana Grover | ^27^Lal Path Labs | archana.grover@lalpathlabs.com |
